# Increased 2-arachidonoyl-*sn-*glycerol levels normalize cortical responses to sound and improve behaviors in *Fmr1* KO mice

**DOI:** 10.1186/s11689-021-09394-x

**Published:** 2021-10-13

**Authors:** Patricia S. Pirbhoy, Carrie R. Jonak, Rashid Syed, Donovan A. Argueta, Pedro A. Perez, Mark B. Wiley, Keon Hessamian, Jonathan W. Lovelace, Khaleel A. Razak, Nicholas V. DiPatrizio, Iryna M. Ethell, Devin K. Binder

**Affiliations:** 1 Division of Biomedical Sciences, School of Medicine, University of California, Riverside, Riverside, CA 92521 USA; 2 Department of Psychology, University of California, Riverside, Riverside, CA 92521 USA

**Keywords:** Electroencephalography, Gamma-band power, Cortical hyperexcitability, Auditory hypersensitivity, 2-Arachidonoyl-*sn*-glycerol, Endocannabinoid modulation

## Abstract

**Background:**

Individuals with Fragile X syndrome (FXS) and autism spectrum disorder (ASD) exhibit an array of symptoms, including sociability deficits, increased anxiety, hyperactivity, and sensory hyperexcitability. It is unclear how endocannabinoid (eCB) modulation can be targeted to alleviate neurophysiological abnormalities in FXS as behavioral research reveals benefits to inhibiting cannabinoid (CB) receptor activation and increasing endocannabinoid ligand levels. Here, we hypothesize that enhancement of 2-arachidonoyl-sn-glycerol (2-AG) in *Fragile X mental retardation 1* gene knock-out (*Fmr1* KO) mice may reduce cortical hyperexcitability and behavioral abnormalities observed in FXS.

**Methods:**

To test whether an increase in 2-AG levels normalized cortical responses in a mouse model of FXS, animals were subjected to electroencephalography (EEG) recording and behavioral assessment following treatment with JZL-184, an irreversible inhibitor of monoacylglycerol lipase (MAGL). Assessment of 2-AG was performed using lipidomic analysis in conjunction with various doses and time points post-administration of JZL-184. Baseline electrocortical activity and evoked responses to sound stimuli were measured using a 30-channel multielectrode array (MEA) in adult male mice before, 4 h, and 1 day post-intraperitoneal injection of JZL-184 or vehicle. Behavior assessment was done using the open field and elevated plus maze 4 h post-treatment.

**Results:**

Lipidomic analysis showed that 8 mg/kg JZL-184 significantly increased the levels of 2-AG in the auditory cortex of both *Fmr1* KO and WT mice 4 h post-treatment compared to vehicle controls. EEG recordings revealed a reduction in the abnormally enhanced baseline gamma-band power in *Fmr1* KO mice and significantly improved evoked synchronization to auditory stimuli in the gamma-band range post-JZL-184 treatment. JZL-184 treatment also ameliorated anxiety-like and hyperactivity phenotypes in *Fmr1* KO mice.

**Conclusions:**

Overall, these results indicate that increasing 2-AG levels may serve as a potential therapeutic approach to normalize cortical responses and improve behavioral outcomes in FXS and possibly other ASDs.

**Supplementary Information:**

The online version contains supplementary material available at 10.1186/s11689-021-09394-x.

## Background

Fragile X syndrome (FXS) is a neurodevelopmental disorder caused by the transcriptional silencing of the *Fragile X mental retardation 1* (*Fmr1*) gene [1], which leads to the loss of the Fragile X Mental Retardation Protein (FMRP) [2]. The loss of FMRP induces a spectrum of molecular [3] and synaptic alterations in structure [4, 5] and function [6–8] that may underlie circuit dysfunction across brain regions [9–12]. Individuals with FXS are phenotypically heterogeneous, but common characteristics include intellectual disability [13], social and communication deficits [14], increased anxiety [15, 16], repetitive behaviors [17], sensory hyperexcitability [18–20], and in some cases, epilepsy [21] [reviewed in [22]].

Electroencephalographic (EEG) studies in human patients with FXS show baseline gamma-band abnormalities and impaired synchronization to auditory stimuli, which are associated with heightened sensory sensitivities and social communication deficits [23, 24]. These biomarkers also correlate with reduced behavioral flexibility, increased distractibility, and cognitive deficits [25]. Remarkably similar EEG findings are observed in rodent models of FXS, the *Fmr1* knock-out (KO) rat [26] and mouse [27–30], where both developing [29, 30] and adult [27, 28] *Fmr1* KO mice exhibit increased gamma-band power in the auditory and frontal cortex compared to wild-type (WT) counterparts. The reproducibility of these neurophysiological measures in replicate studies and FXS rodent models provides a unique opportunity to assess the efficacy of pharmacological interventions. In this study, EEG assessment was used to test the efficacy of an endocannabinoid (eCB)-positive modulator, JZL-184, which inhibits monoacylglycerol lipase (MAGL) [31, 32], a hydrolase involved in 2-arachidonoyl-*sn-*glycerol (2-AG) degradation [33], as a potential therapeutic for FXS.

The use of cannabis and/or cannabis-related products has risen over the years for various disorders, including but not limited to epilepsy [34], multiple sclerosis [35], and autism spectrum disorders (ASDs) [36]. Evidence implicating beneficial effects of cannabinoid modulation in FXS phenotypes includes effects on cognition [37, 38], anxiety [36, 39], restlessness [36], nociception [40], and audiogenic seizure susceptibility [39]. Yet, it is unclear how eCB system modulation can be selectively targeted to achieve the multifaceted benefits of cannabinoids. One theory of eCB system dysfunction in FXS is attributed to alterations in metabotropic glutamate receptor 5 (mGluR5) and diacylglycerol lipase-alpha (DAGL-alpha) coupling [41], specifically termed the “eCB signalosome” [42, 43]. The altered coupling leads to disruption of mGluR5-mediated production of 2-arachidonoyl-*sn-*glycerol (2-AG) in the ventral striatum and prefrontal cortex of FXS mouse models [43]. Furthermore, enhancement of 2-AG was shown to normalize synaptic deficits and behavioral abnormalities [43].

To test the hypothesis that increasing levels of 2-AG normalizes abnormal EEG patterns in *Fmr1* KO mice, we employed a selective and irreversible MAGL inhibitor to increase endogenous 2-AG levels. JZL-184 was chosen based on its ability to inhibit MAGL [31, 32] and use in previous FXS studies [43]. To validate the ability of JZL-184 to increase brain levels of 2-AG, we employed lipidomic analysis in conjunction with various doses and time points post-administration of JZL-184. This enabled the identification of a dose and time point that significantly increased 2-AG levels to test EEG outcomes. EEG recordings were performed at 4 h and 1 day post-treatment using a 30-channel multielectrode array (MEA). Lipidomic analysis showed that JZL-184 treatment had dose- and time-dependent effects on 2-AG brain levels in the auditory cortex, and this correlated with salutary effects on EEG indices and behavior in *Fmr1* KO mice. Overall, these results indicate that eCB system modulation, specifically increasing levels of 2-AG, may serve as a promising therapeutic to ameliorate cortical hyperexcitability and anxiety/hyperactivity phenotypes in FXS and ASD-related disorders.

## Methods

### Ethics statement

All experiments and animal care/use protocols were approved by the Institutional Animal Care and Use Committee at the University of California, Riverside, and were carried out in accordance with NIH “Guide for the Care and Use of Laboratory Animals.”

### Animals

Experimental animals were C57BL/6 wild type (WT, RRID:IMSR JAX:000,664) and *Fragile X mental retardation gene-1* (*Fmr1*) knock-out (KO) mice (RRID: IMSR_JAX:003,025, The Jackson Laboratory) obtained from Jackson Laboratories. Mice were maintained in an AAALAC accredited facility in 12-h light/dark cycles, and food and water were available ad libitum. Age-matched WT and *Fmr1* KO male mice were used at postnatal day (P) 60–90 for lipidomic analysis (*N* = 78). WT and *Fmr1* KO male littermates were used at P70–85 for EEG recording (*N* = 61, excluded post-surgery *N* = 7) and P60–65 for behavior (*N* = 56). All genotypes were confirmed with the analysis of tail samples by Transnetyx (Cordova, TN, USA), using real-time PCR to probe for WT (fmr + / + forward: 5′-TGT GAT AGA ATA TGC AGC ATG TGA-3′) or KO (fmr-/-forward: 5′CAC GAG ACT AGT GAG ACG TG-3′) target sequence in each sample as described previously [30].

### Treatment

The MAGL inhibitor, JZL-184 (Tocris, Cat. 3836), in saline (85%) containing 7.5% DMSO, 7.5% Tween 80, or vehicle (7.5% DMSO, 7.5% Tween-80 in 85% saline) was administered intraperitoneally (i.p., 4, 8, and 16 mg/kg) to male WT and *Fmr1* KO mice at P60–90. Animals were assigned treatment based on simple randomization. To ensure that the experimenter was blind to treatment for experimental procedures, one experimenter prepared drugs and assigned a numerical identifier to each treatment/animal, and a second experimenter administered the treatment and was blinded to each assigned treatment. For lipidomic analysis, naive mice received a single treatment of JZL-184 (4, 8, and 16 mg/kg) or vehicle injections at P60–90, and brains were removed 4 h or 24 h post-injection for lipidomic analysis. For EEG recordings, mice were recorded prior to treatment (PRE, non-injected animals) and then received a single treatment of JZL-184 (8 mg/kg) or vehicle and were subjected to EEG recording at 4 h and 1 day post-treatment. For behavioral testing, naive mice received a single treatment of JZL-184 (8 mg/kg) or vehicle injections at P60–65 and were subjected to behavioral testing at 4 h post-injection.

### Endocannabinoid lipid extract

Animals were euthanized with isoflurane at the time of collection. The brain was quickly removed and washed in ice-cold phosphate-buffered saline (PBS), on a glass petri dish on ice, and the auditory cortex removed as previously described [30]. Samples were snap-frozen in 2-methylbutane followed by liquid nitrogen and then stored at − 80 °C pending analysis. Lipid extract procedures were done as previously described [44–46]. Frozen tissues were weighed and then homogenized in 1 mL methanol solution containing the internal standards, [^2^H_5_]-2-AG (500 pmol), [^2^H_4_]-AEA (1 pmol), and [^2^H_4_]-OEA (10 pmol) (Cayman Chemicals, Ann Arbor, MI). Lipids were extracted with chloroform (2 mL) and washed with water (1 mL). Organic phases were collected and separated by open-bed silica gel column chromatography. Eluate was gently dried under nitrogen stream (99.998% pure) and resuspended in 0.2 mL methanol:chloroform (1:1), with 1 μL injection for analysis by ultra-performance liquid chromatography coupled to tandem mass spectrometry (UPLC-MS/MS).

### Measurement of FAEs/MAGs

Data were collected as described previously [44–46] using an Acquity I Class UPLC system coupled to a Xevo TQ-S Mass Spectrometer (Waters, Milford, MA, USA) with accompanying electrospray ionization (ESI) sample delivery. Lipids were separated on an Acquity UPLC BEH C_18_ Column (2.1 × 50 mm i.d., 1.7 μm, Waters) with inline Acquity guard column (UPLC BEH C_18_ VanGuard Pre-column; 2.1 × 5 mm i.d., 1.7 μm, Waters) and eluted by a gradient of methanol in water (0.25% acetic acid, 5 mM ammonium acetate) according to the following gradient at a flow rate of 0.4 mL per min: 80% methanol 0.0–0.5 min, 80 to 100% methanol 0.5–2.5 min, 100% methanol 2.5–3.0 min, 100–80% methanol 3.0–3.1 min, and 80% methanol 3.1–4.5 min. Column temperature was maintained at 40 °C and samples were maintained in the sample manager at 10 °C. Argon (99.998%) was used as a collision gas. MS detection was in positive ion mode and capillary voltage set at 0.1 kV. Cone voltage and collision energy were as follows, respectively: 2-AG = 30v, 12v; [^2^H_5_]-2-AG = 25v, 44v; DHAG = 34v, 14v; 2-OG 42v, 10v; 2-LG = 30v, 10v; anandamide (AEA) = 30v, 14v; [^2^H_4_]-AEA = 26v, 16v; OEA = 28v, 16v; [^2^H_4_]-OEA = 48v, 14v; DHEA = 30v, 50v. Lipids were quantified using a stable isotope dilution method detecting protonated adducts of the molecular ions [M + H]^+^ in the multiple reaction monitoring mode (MRM). Acyl migration from 2-AG to 1-AG is known to occur; thus, all reported values for 2-AG represent the sum of 2-AG and 1-AG. Extracted ion chromatograms were used to quantify 2-AG (*m*/*z* = 379.3 > 287.3), DHAG (*m*/*z* = 403.3 > 311.1), 2-OG (*m*/*z* = 357.4 > 265.2), 2-LG (*m*/*z* = 355.3 > 263.3), AEA (*m*/*z* = 348.3 > 62.0), OEA (*m*/*z* = 326.4 > 62.1), and DHEA (*m*/*z* = 372.4 > 91.02) with [^2^H_5_]-2-AG (*m*/*z* = 384.3 > 93.4), [^2^H_4_]-AEA (*m*/*z* = 352.3 > 66.1), and [^2^H_4_]-OEA (*m*/*z* = 330.3 > 66.05) used as internal standards. Analytes: 2-AG, 2-docosahexaenoylglycerol (DHAG), 2-lineolyeoylglycerol (2-LG), 2-oleoylglycerol (2-OG), AEA, docosahexaenoyl ethanolamide (DHEA), oleoylethanolamide (OEA).

### Multielectrode array (MEA) implantation

Surgical procedures were performed as described in detail previously [27, 47]. Briefly, mice were anesthetized with isoflurane inhalation (0.2–0.5%) and given an intraperitoneal (i.p.) injection of ketamine and xylazine (K/X 100/20 mg/kg). Toe pinch reflex was used to measure anesthetic state every 10 min throughout the surgery, and supplemental doses, no more than half of the original dose of K/X, were administered as needed. Mice were aseptically prepared for surgery and placed in a stereotaxic frame (model 930; Kopf, CA). Artificial tear ointment (Henry Schein, 01,169,568,321) was applied to the eyes to prevent drying. Once the mouse was anesthetized, a midline sagittal incision was made along the scalp to expose the skull. A cotton-tip applicator was used to remove the periosteum from the skull and to clean the skull with saline. A surgical marker was used to mark bregma. A Foredom dental drill was used to drill three holes 1 mm in diameter in the skull overlying the left frontal cortex, left cerebellum, and right cerebellum. One-millimeter stainless steel screws (PlasticsOne, 00–96 X 1/16) were advanced into the drilled holes until secure. Special care was taken not to advance the screws beyond the point of contact with the dura. The probe grounding wire was placed in the nuchal musculature, and the 30-channel MEA probe was placed on the skull surface, carefully aligning the “ + ” in the center of the probe with bregma. Saline was added to the top of the probe to aid in adherence to the skull surface and allowed to dry. A 4–0 silk tie was used to secure the probe ribbon between the two cerebellum screws, and Teflon/plastic wrap was placed on top of the probe secured by the three screws. Dental cement was applied around the screws, on the base of the cotton-tip applicator post, and the Teflon/plastic wrap covering the probe. A waterproof medical tape was used to secure the cotton-tip applicator to the probe connector.

Postoperative care included the topical application of a triple antibiotic ointment along the edges of the dental cement followed by two subcutaneous injections of buprenorphine (0.1 mg/kg; Reckitt & Colman, 5,053,624), one immediately after surgery and one 8–12 h after surgery. Mice were placed on a heating pad to aid recovery from anesthesia, and additional doses of buprenorphine were administered every 8 h for continuous analgesia during the first 48 h after surgery. Mice were individually housed with nesting material (Ancare, NES3600) for environmental enrichment and monitored daily until the day of EEG recordings, which allowed 3–5-day recovery post-surgery before recording.

### Electrophysiology

Resting and auditory event-related potential recordings were obtained using the SmartBox (NeuroNexus) from awake and freely moving mice as previously described [27, 47]. Mice were habituated for 20 min in an anechoic foam-lined sound-attenuating chamber (Gretch-Ken Industries Inc., OR). A head-stage and tether were connected to the probe post of the mouse skull under brief isoflurane anesthesia. The tether was connected to a commutator located directly above the cage. Mice were then allowed to habituate to being connected to the tether for an additional 20 min before EEG recordings were obtained.

The SmartBox acquisition system was connected to the commutator to which the animal was attached allowing recordings from awake and freely moving mice. The acquisition hardware filters were set to pass signals between (0.5 and 500 Hz) and the data were sampled at a rate of 1250 Hz. Sound delivery was synchronized with EEG recording using a TTL pulse to mark the onset of each sound in a train. Resting EEGs were recorded for 5 min (during this time, no auditory stimuli were presented), followed by recordings in response to auditory stimulation.

### Acoustic stimulation

Acoustic stimulation paradigms were similar to those previously used in *Fmr1* KO mice [27, 28, 30, 48], which is analogous to work in humans with FXS. All experiments were conducted in a sound-attenuated chamber lined with anechoic foam (Gretch-Ken Industries Inc., OR). Acoustic stimuli were generated using RPvdsEx software and RZ6 hardware (Tucker-Davis Technologies, FL) and presented through a free-field speaker (MF1 Multi-Field Magnetic Speaker; Tucker-Davis Technologies, FL) located 30 cm directly above the cage. Sound pressure level (SPL) was modified using programmable attenuators in the RZ6 system. The speaker output was ~ 70 dB SPL at the floor of the recording chamber with fluctuation of ± 3 dB for frequencies between 5 and 35 kHz as measured with a ¼ in Bruel and Kjaer microphone.

A chirp-modulated signal (henceforth “chirp”) to induce synchronized oscillations in EEG recordings was used to quantify the fidelity of responses to time-varying stimuli. The chirp is a 2-s broadband noise stimulus which is amplitude modulated (100% modulation depth) by a sinusoid whose frequencies increase (Up-chirp) or decrease (Down-chirp) linearly in the 1–100-Hz range [49–51]. The chirp facilitates a rapid measurement of transient oscillatory response (delta to gamma frequency range) to auditory stimuli of varying frequencies and can be used to compare the oscillatory response in different groups in clinical and pre-clinical settings. Inter-trial phase coherence (ITPC) analysis (phase-locking factor across trials) can then be used to determine the ability of the neural generator to synchronize oscillations to the frequencies present in the stimulus [52]. The chirp stimulus may be preferable over the traditional steady-state stimulus in studies of children with neurodevelopmental disorders, as it can quickly and efficiently measure multiple modulation frequencies in a shorter period of time [25]. The chirp stimulus and ITPC measurements show similar deficits in temporal processing in both humans with FXS and the *Fmr1* KO mice.

To avoid onset responses contaminating phase locking to the amplitude modulation of the chirp, the stimulus was ramped in sound level from 0 to 100% over 1 s (rise time), which then smoothly transitioned into chirp modulation of the noise. Chirp trains were presented 300 times with an interval between each train randomly varied between 1 and 1.5 s. The total duration of the chirp stimulation EEG recording was 25 min.

### Data analysis

All EEG data analysis was performed as described previously [27]. Briefly, data analyses were done using a combination of Analyzer 2.1 software (Brain Vision Inc.), MATLAB, and SPSS. Data were extracted from the SmartBox files and saved in a file format compatible with Analyzer 2.1 software. Data were first down-sampled to 625 Hz, and a 60-Hz notch filter was used to remove residual line noise from recordings. EEG artifact rejection was performed using a semi-automatic protocol in Analyzer 2.1 software. Several criteria were used to search for artifacts, including amplitude, gradient, and max–min. Less than 30% of data were rejected due to artifacts from any single mouse. If more than 30% of the data was rejected, the animal was excluded from analysis (resting baseline/chirp: WT PRE, ***N*** = 3; *Fmr1* KO PRE, ***N*** = 5; JZL-treated *Fmr1* KO, ***N*** = 2 (4 h) and ***N*** = 7 (1 day); JZL-treated WT, ***N*** = 0 (4 h) and ***N*** = 2 (1 day); vehicle-treated *Fmr1* KO, ***N*** = 3 (4 h) and ***N*** = 7 (1 day); vehicle-treated WT, ***N*** = 4 (4 h) and ***N*** = 3 (1 day)). An outlier test was also performed using the Prism Outlier Calculator for resting power ratio calculations. If an animal was a significant outlier, it was removed from the analysis (*N* = 1).

#### Resting EEG and sound-evoked response analyses

Five minutes of EEG data (no auditory stimulus) was divided into 1-s segments, and fast Fourier transform (FFT) was calculated on each segment using 0.5-Hz bins, using a Hanning window, with no overlap, and then average power density (μV^2^/Hz) was calculated for each mouse from 1 to 100 Hz as previously described [27]. Power was then further binned into standard frequency bands: delta (1–4 Hz), theta (4–8 Hz), alpha (8–13 Hz), beta (13–30 Hz), low gamma (30–55 Hz), and high gamma (65–100 Hz). We compared genotype or treatment group mean differences on six bands per region: left frontal (LF), right frontal (RF), left medial (LM), right medial (RM), left temporal (LT), and right temporal (RT). The spectral bands used are based on a number of previous studies on FXS in both mice [53] and humans [20, 23, 24].

Responses to chirp trains were analyzed using Morlet wavelet analysis as previously described [27]. Chirp trains were segmented into windows of 500 ms before chirp onset to 500 ms after the end of the chirp sound (a total of 3 s because each chirp was 2 s in duration). EEG traces were processed with Morlet wavelets from 1 to 100 Hz using complex number output (voltage density, μV/Hz) for inter-trial phase coherence (ITPC) calculations and baseline corrected non-phase-locked single-trial power (induced power). Wavelets were run with a Morlet parameter of 10 as this gave the best frequency/power discrimination. This parameter was chosen since studies in humans found the most robust difference around 40 Hz, where this parameter is centered [23].

To measure phase synchronization at each frequency across trials, ITPC was calculated. The equation used to calculate ITPC is:

$$ITPC(f,t)=\frac{1}{n}\sum_{k=1}^{n}\frac{{F}_{k}(f,t)}{{|F}_{k}(f,t)|}$$

where *f* is the frequency, *t* is the time point, and *ĸ* is the trial number. Thus, *F*_*k*_ (*f*,*t*) refers to the complex wavelet coefficient at a given frequency and time for the *ĸ*th trial. There were no less than 215 trials (out of 300) for any given mouse after segments containing artifacts were rejected.

#### Statistical analysis

All statistical analyses were performed as described previously [27, 28, 48]. The term “resting” is used to indicate EEGs recorded without any specific auditory stimuli. The data were analyzed using a mixed model with multivariate analysis of frequency for each separate region (left/right frontal, left/right medial, left/right temporal). Data was analyzed using a two-way ANOVA. Data are often expressed and plotted as a ratio of control group values to gauge relative differences in various factors using the same scale. Prism 9 software (GraphPad Software, San Diego, USA) was used for statistical analysis.

Statistical group comparisons of chirp responses (ITPC) were quantified by wavelet analysis using MATLAB (MATLAB, RRID:SCR_001622). The analysis was conducted by binning time into 625 parts and frequency into 100 parts, resulting in a 100 × 625 matrix. Non-parametric cluster analysis was used to determine contiguous regions in the matrix that were significantly different from a distribution of 1000 randomized Monte Carlo permutations based on previously published methods [54]. Briefly, if the cluster size of the real genotype assignments (both positive and negative direction, resulting in a two-tailed alpha of *p* = 0.025) was larger than 97.25% of the random group assignments, those clusters were considered significantly different between groups. This method avoids statistical assumptions about the data and corrects for multiple comparisons.

In all cases, *p* < 0.05 was considered significant for ANOVA and Student’s *t*-tests. Where *t*-tests were performed, *r* was calculated as an effect size. When multiple comparisons for ANOVA were made, data were analyzed on each factor for simple effects and corrected using Bonferroni adjustments. In all cases, means are reported, and the standard error of the mean (SEM) was used. For all analyses, “N” = the number of animals.

Power analysis was performed to calculate sample size for each experiment, and for EEG, power analysis estimated an *N* of 10 mice per group. The variance in the power analysis estimate was determined using previously published studies using similar techniques [EEG [27–29]].

### Behavior

#### Open-field (OF) test

Behavioral tests were performed as described previously [30, 48, 55]. Briefly, anxiety-like behavior was tested (*N* = 9–12 mice per group) by quantifying their tendency to travel to the center of an open field and time spent in thigmotaxis [56, 57]. A 43 cm × 43 cm open-field arena with 43-cm-high walls was constructed from clear acrylic sheets. The open-field arena was placed in a brightly lit room, and one mouse at a time was placed in a corner of the open field and allowed to explore for 10 min while being recorded with digital video from above. The floor was cleaned with 3% acetic acid, 70% ethanol, and water between tests to eliminate odor trails. Mice were tested between 8:00 AM and 5:00 PM, and this test was always performed prior to the elevated plus maze by the same experimenter. The arena was subdivided into a 4 × 4 grid of squares, with the middle of the grid defined as the center. A line 4 cm from each wall was added to measure thigmotaxis. Locomotor activity was scored by the analysis of total line crosses and speed as described previously with some modifications [56] using TopScan Lite Software (Clever Sys., Inc., Reston, VA 201,090, USA). A tendency to travel to the center (total number of entries into large and small center squares) and the time in thigmotaxis were used as an indicator of anxiety-like behaviors using TopScan Lite software (CleverSys Inc). The analysis was performed on the first 5-min interval of the total 10-min exploration duration. Assessments of the digital recordings were performed by an experimenter blind to the treatment groups using TopScan Lite Software or manual quantification of rearing, stretch-attend posture, defecation, and grooming bouts. Statistical analysis was performed with an unpaired two-way ANOVA using GraphPad Prism 9 software. Data represent the mean ± standard error of the mean (SEM). An outlier test was also performed using the Prism Outlier Calculator for behavioral measures. If an animal was a significant outlier, it was removed from the analysis (vehicle-treated *Fmr1* KO, *N* = 1; JZL-184-treated *Fmr1* KO, *N* = 1).

#### Elevated plus maze (EPM)

Behavioral tests on the EPM were performed as described previously [30, 48, 55]. The EPM consisted of four arms in a plus configuration. Two opposing arms had 15-cm tall walls (closed arms), and two arms were without walls (open arms). The entire maze sat on a stand 1 m above the floor. Each arm measured 30 cm long and 10 cm wide. Mice were allowed to explore the maze for 10 min while being recorded by digital video from above. The maze was wiped with 3% acetic acid, 70% ethanol, and water between each test to eliminate odor trails. TopScan Lite software was used to measure the percent of time spent in open arms and speed. The time spent in the open arm was used to evaluate anxiety-like behavior. The velocity and total arm entries were measured to evaluate overall locomotor activity. The analysis was performed on the first 5-min interval of the total 10-min exploration duration. Video assessment and statistical analysis were performed as described above for the OF test. An outlier test was also performed using the Prism Outlier Calculator for behavioral measures. The outlier test resulted in the removal of one JZL-184-treated WT mouse and one JZL-184-treated *Fmr1* KO mouse.

## Results

In the current study, we examined whether increasing the levels of the eCB receptor ligand, 2-AG, normalizes neural correlates of auditory hypersensitivity and behavioral deficits in *Fmr1* KO mice. To do this, mice were treated with the MAGL inhibitor, JZL-184, and control animals were treated with vehicle. To confirm increased levels of 2-AG following JZL-184 treatment, *Fmr1* KO and WT mice were treated with 4, 8, or 16 mg/kg JZL-184 or vehicle, and the auditory cortex was dissected and processed for lipidomic analysis. A second group of mice was subjected to 30-channel MEA EEG recording to quantify resting baseline power spectral density and sound-evoked responses prior to treatment (PRE, non-injected animals), 4 h and 1 day post-treatment with 8 mg/kg JZL-184 or vehicle. A third group of mice was used to assess hyperactivity and anxiety-like behaviors using the open field and the elevated plus-maze tests. Only adult (P60–90) male mice were used in this study.

### JZL-184 treatment increases levels of 2-AG in the cortex of *Fmr1* KO and WT mice

**Fig. 1:**
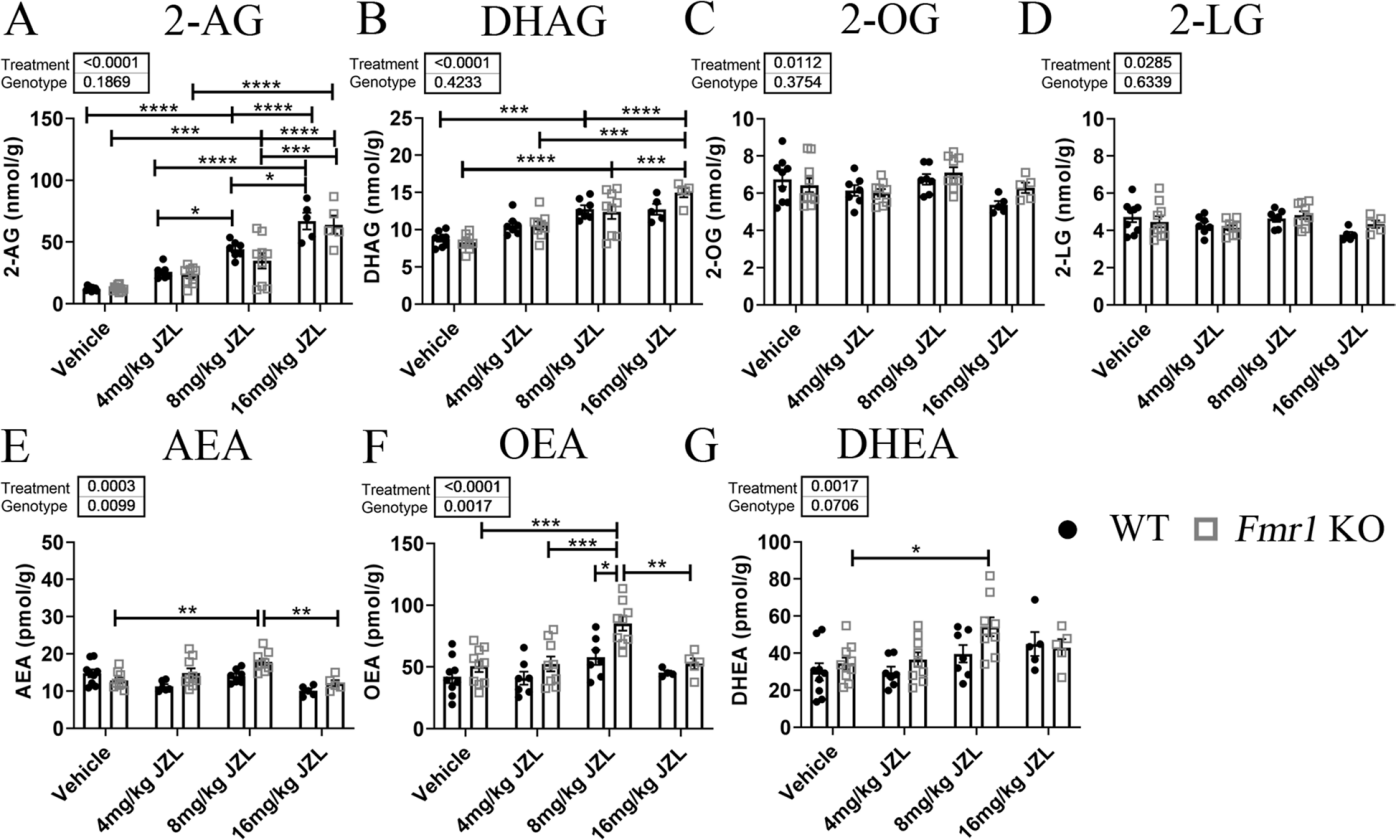
Effects of JZL-184 treatment on mouse cortical lipidomic profile. Mouse brain levels of 2-arachidonoyl-*sn-*glycerol (2-AG, **A**), docosahexaenoylglycerol (DHAG, **B**), 2-oleoylglycerol (2-OG, **C**), 2-linoleoyl glycerol (2-LG, **D**), anandamide (AEA, **E**), oleoylethanolamide (OEA, **F**), and docosahexaenoyl ethanolamide (DHEA, **G**) 4 h post-treatment in *Fmr1* KO and WT mice treated with 4, 8, or 16 mg/kg JZL-184 or vehicle. Values represent means per group and error bars indicate the standard error of the mean (SEM). Statistical analysis was performed using the two-way ANOVA for all comparisons. *p < 0.05, **p < 0.01, ***p < 0.001, ****p < 0.0001. Vehicle WT, *N* = 8–9; 4 mg/kg JZL-184 WT, *N* = 6–7; 8 mg/kg JZL-184 WT, *N* = 7; 16 mg/kg JZL-184 WT, *N* = 4–5; vehicle *Fmr1* KO, *N* = 9–10; 4 mg/kg JZL-184 *Fmr1* KO, *N* = 9; 8 mg/kg JZL-184 *Fmr1* KO, *N* = 9; 16 mg/kg JZL-184 *Fmr1* KO, *N* = 5. Excluded: AEA, 4 mg/kg JZL-184 WT, *N* = 1; vehicle *Fmr1* KO, *N* = 1. OEA, 16 mg/kg JZL-184 WT, *N* = 1. DHAG, vehicle WT, *N* = 1

In the current study, we examined whether increasing the levels of the eCB receptor ligand, 2-AG, normalizes neural correlates of auditory hypersensitivity in *Fmr1* KO mice. To confirm that MAGL inhibition with JZL-184 results in increased levels of 2-AG in the auditory cortex, animals were injected with 4, 8, or 16 mg/kg JZL-184 or vehicle and brains were collected either 4 h or 1 day post-injection for lipidomic analysis. Lipids were extracted from cortical tissue and analyzed using ultra-performance liquid chromatography coupled to tandem mass spectrometry (UPLC-MS/MS). Assessment of 2-AG levels 4 h post-treatment using the two-way ANOVA revealed a significant main effect of treatment (*F*(3,53) = 62.38, *p* < 0.0001), but no significant interaction (*F*(3,53) = 0.64, *p* = 0.59) or genotype effect (*F*(1,53) = 1.79, *p* = 0.19). Post hoc comparisons showed a significant increase in 2-AG levels with 8–16 mg/kg JZL-184 in WT (4 mg/kg, *p* = 0.34; 8 mg/kg, *p* < 0.0001; 16 mg/kg, *p* < 0.0001) and *Fmr1* KO (4 mg/kg, *p* = 0.88; 8 mg/kg, *p* = 0.0006, 16 mg/kg, *p* < 0.0001) mice compared to vehicle controls (Fig. 1A).

Treatment with JZL-184 also increased levels of docosahexaenoylglycerol (DHAG) in the cortex of JZL-184-treated *Fmr1* KO (4 mg/kg JZL-184, *N* = 9, 10.6 ± 0.53 nmol/g; 8 mg/kg JZL-184, *N* = 9, 12.4 ± 0.96 nmol/g; 16 mg/kg JZL-184, *N* = 5, 15.0 ± 0.62 nmol/g) and WT mice (4 mg/kg JZL-184, *N* = 7, 10.6 ± 0.51 nmol/g; 8 mg/kg JZL-184, *N* = 7, 12.8 ± 0.51 nmol/g; 16 mg/kg JZL-184, *N* = 5, 12.7 ± 0.73 nmol/g) 4 h post-treatment compared to vehicle-treated *Fmr1* KO (vehicle, *N* = 10, 8.3 ± 0.31 nmol/g) and WT mice (vehicle, *N* = 8, 8.7 ± 0.36 nmol/g; Fig. 1B). Statistical analysis using the two-way ANOVA revealed a significant main effect of treatment (*F*(3,52) = 28.21, *p* < 0.0001), but no significant interaction (*F*(3,52) = 1.64, *p* = 0.19) or genotype effect (*F*(1,52) = 0.65, *p* = 0.42). Post hoc comparisons showed a significant increase in DHAG levels specifically with 8 mg/kg and 16 mg/kg JZL-184 for WT mice (4 mg/kg, *p* = 0.96; 8 mg/kg, *p* = 0.0006; 16 mg/kg, *p* = 0.003) and *Fmr1* KO (4 mg/kg, *p* = 0.12; 8 mg/kg, *p* < 0.0001, 16 mg/kg, *p* < 0.0001) mice compared to vehicle controls. Statistical analysis of 2-oleoylglycerol (2-OG) or 2-linoleoyl glycerol (2-LG) using the two-way ANOVA revealed a significant main effect of treatment for both 2-OG and 2-LG (2-OG, *F*(3,53) = 4.08, *p* = 0.01; 2-LG, *F*(3,53) = 3.26, *p* = 0.03), but no significant interaction (2-OG, *F*(3,53) = 1.25, *p* = 0.30; 2-LG, *F*(3,53) = 1.09, *p* = 0.36) or genotype effect (2-OG, *F*(1,53) = 0.80, *p* = 0.38; 2-LG, *F*(1,53) = 0.23, *p* = 0.63). Post hoc comparisons revealed no significant pairwise comparisons (Fig. 1C and D, respectively).

JZL-184 is a selective MAGL inhibitor and it is not expected to alter levels of AEA, which is hydrolyzed by the fatty acid amid hydrolase (FAAH). Interestingly, lipidomic analysis of fatty acid ethanolamides (FAEs) revealed significant changes in *Fmr1* KO mice (Fig. 1E–G). Statistical analysis with a two-way ANOVA of AEA levels 4 h post-treatment revealed a significant main effect of treatment (*F* (3,51) = 7.66, *p* = 0.0003) and genotype (*F*(1,51) = 7.18, *p* = 0.009) and a significant interaction (*F*(3,51) = 4.41, *p* = 0.008). Post hoc comparisons showed a significant increase in AEA levels in 8 mg/kg JZL-184-treated *Fmr1* KO mice compared to vehicle-treated *Fmr1* KO mice (*p* = 0.004) and a significant decrease in 16 mg/kg JZL-184-treated *Fmr1* KO mice compared to 8 mg/kg JZL-184-treated *Fmr1* KO mice (Fig. 1E, p = 0.006). Statistical analysis of oleoylethanolamide (OEA) and docosahexaenoyl ethanolamide (DHEA) levels 4 h post-JZl-184 treatment with a two-way ANOVA revealed a significant main effect of treatment (OEA, *F*(3,52) = 10.07, *p* < 0.0001; DHEA, *F*(3,53) = 5.79, *p* = 0.002), a significant genotype effect for OEA (*F*(1,52) = 11.01, *p* = 0.002) but not DHEA (*F*(1,53) = 3.40, *p* = 0.07) and no significant interaction for both OEA and DHEA (OEA, *F*(3,52) = 1.42, *p* = 0.25; DHEA, *F*(3,53) = 1.01, *p* = 0.40). Post hoc comparisons revealed a significant increase in OEA levels in 8 mg/kg *Fmr1* KO mice compared to vehicle-treated *Fmr1* KO mice and a significant decrease in 16 mg/kg JZL-184-treated *Fmr1* KO mice compared to 8 mg/kg JZL-184-treated *Fmr1* KO mice (Fig. 1F, p = 0.009). Post hoc comparisons also revealed a significant genotype difference in OEA levels between 8 mg/kg JZL-184-treated *Fmr1* KO and 8 mg/kg JZL-184-treated WT mice (*p* = 0.02). Post hoc comparisons of DHEA levels revealed a significant increase in 8 mg/kg JZL-184-treated *Fmr1* KO compared to vehicle-treated *Fmr1* KO mice (Fig. 1G, p = 0.03; see Supplementary Table S1 for a summary of statistical results, Additional file 1).

Assessment of 2-AG, DHAG, 2-OG, AEA, OEA, and DHEA 1 day post-treatment revealed no significant changes in lipid levels between vehicle and 8 mg/kg JZL-184-treated *Fmr1* KO or WT mice (see Supplementary Figure S1 and Supplementary Table S2 for statistical comparisons, Additional file 1).

Overall, lipidomic results confirm an increase in 2-AG levels along with an increase in DHAG, AEA, OEA, and DHEA in *Fmr1* KO mice treated with 8 mg/kg JZL-184 compared to vehicle-treated *Fmr1* KO mice. In WT mice, there was a significant increase selectively in 2-AG and DHAG levels in 8 mg/kg JZL-184-treated mice compared to vehicle-treated WT mice. Given these results, experimental procedures were done using an 8 mg/kg JZL-184 dose at 4 h post-treatment.

### Effects of JZL-184 treatment on enhanced resting-state EEG gamma power in *Fmr1* KO mice

**Fig. 2:**
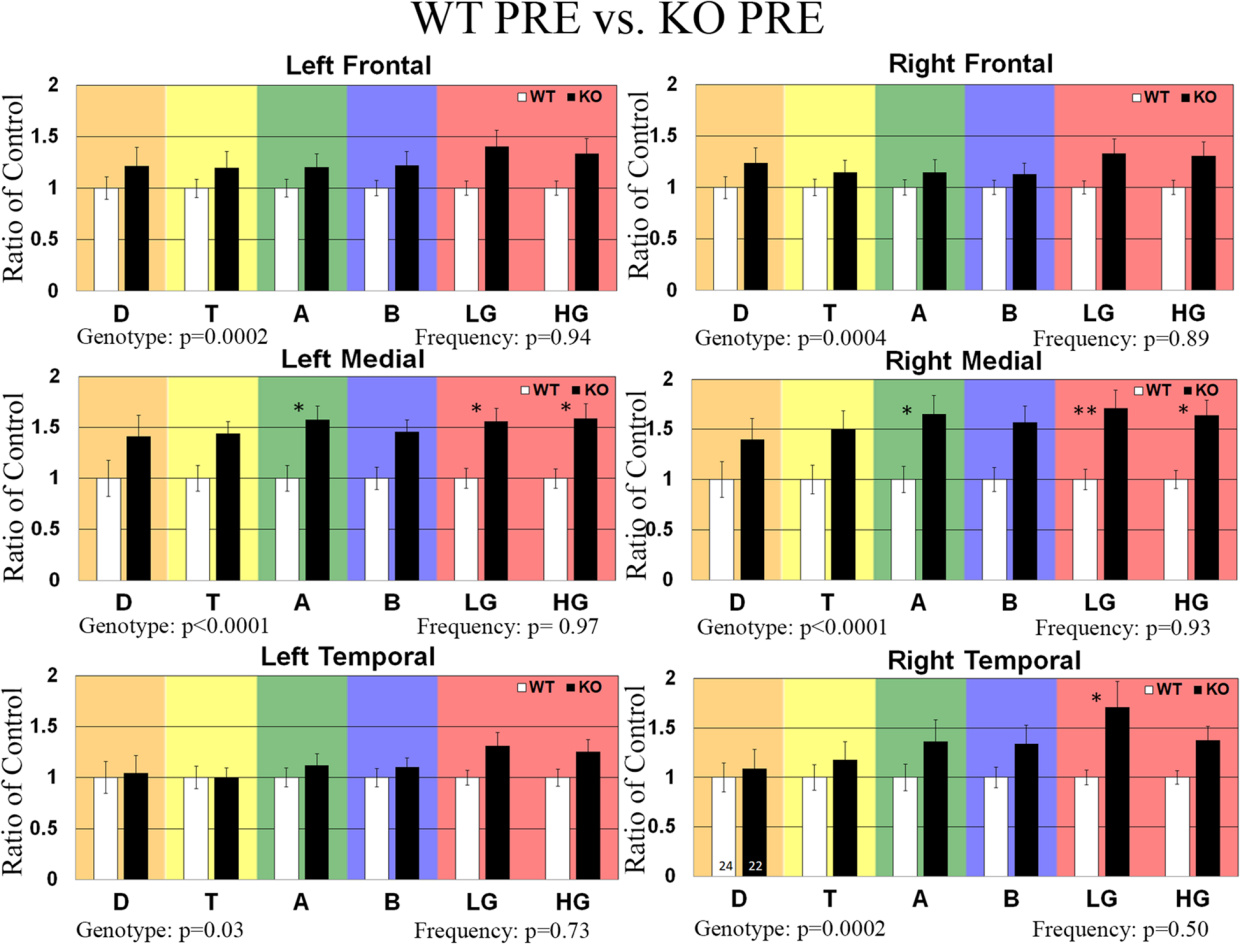
Effects of JZL-184 treatment on resting EEG baseline in non-injected WT and *Fmr1* KO mice. Graphs show average spectral power for EEG resting baseline in left/right frontal, medial, and temporal regions of WT (*N* = 24) and *Fmr1* KO (*N* = 22) mice prior to treatment (PRE). Statistical analysis was performed using the two-way ANOVA for all comparisons. Values represent means per group and error bars represent the standard error of the mean (SEM). *p < 0.05, **p < 0.01, ***p < 0.001, ****p < 0.0001. Abbreviations: D, delta; T, theta; A, alpha; B, beta; LG, low gamma; HG, high gamma

**Fig. 3:**
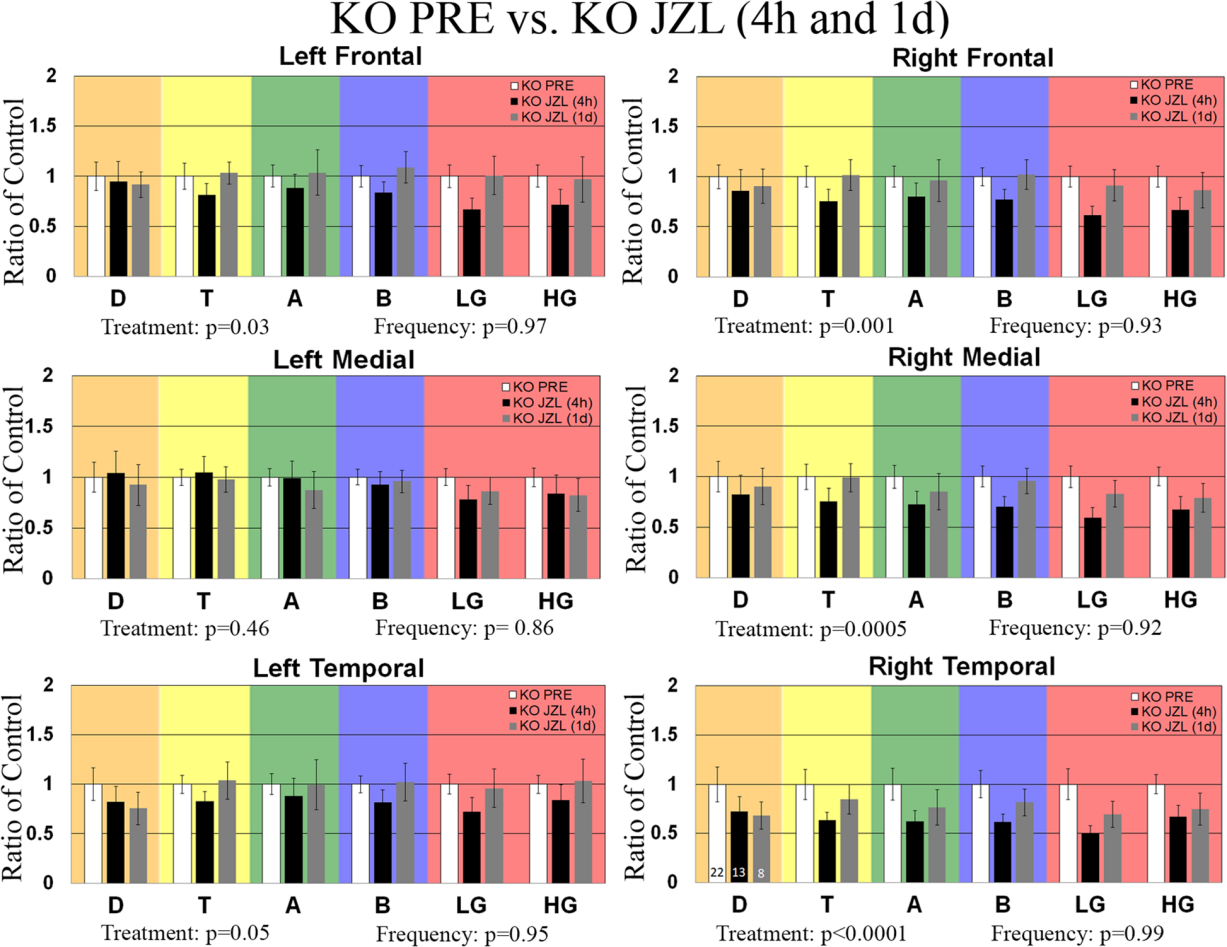
Effects of JZL-184 treatment on resting EEG baseline in *Fmr1* KO mice. Graphs show average spectral power for EEG resting baseline in left/right frontal, medial, and temporal regions of *Fmr1* KO mice treated with 8 mg/kg JZL-184 (*N* = 13) 4 h and 1 day (*N* = 8) post-treatment as a ratio of *Fmr1* KO mice prior to treatment (PRE, *N* = 22). Statistical analysis was performed using the two-way ANOVA for all comparisons. Values represent means per group and error bars represent the standard error of the mean (SEM). *p < 0.05, **p < 0.01, ***p < 0.001, ****p < 0.0001. Abbreviations: D, delta; T, theta; A, alpha; B, beta; LG, low gamma; HG, high gamma

Previously, we have shown that adult and developing *Fmr1* KO mice exhibit increased resting-state baseline gamma power using a 2-channel EEG electrode [28–30] and more recently using a 30-channel MEA probe [27], a phenotype that is also observed in humans with FXS [23, 24]. In this study, we use this EEG biomarker to assess the efficacy of the eCB system modulator, JZL-184, as a potential therapeutic for FXS. To do this, adult male mice were implanted with a 30-channel MEA probe [47]. Mice recovered for 3–5 days, and baseline EEG activity was recorded prior to treatment (PRE). Baseline EEG data (in the absence of auditory stimulation) were collected for 5 min and was divided into 1-s segments for spectral analysis. Resting EEG raw power (μV^2^/Hz) was calculated in six regions (left/right frontal (LF/RF), left/right medial (LM/RM), and left/right temporal (LT/RT)) by analyzing all frequency bands during the entire 5-min resting period. The average power spectral densities for the six regions were also calculated and normalized to a control group to determine relative changes in power across frequency bands. A comparison of power spectral densities of *Fmr1* KO as a ratio of WT mice prior to treatment (non-injected animals) is shown in Fig. 2. Results show a significant increase in gamma-band frequencies in medial and temporal regions. Following the PRE EEG recording, mice received a single intraperitoneal injection of the MAGL inhibitor at 8 mg/kg JZL-184 or vehicle. Post-treatment EEG recordings were collected in the same manner as PRE baseline EEG activity in the same animals 4 h and 1 day post-treatment to assess the efficacy of JZL-184 to normalize resting EEG oscillation patterns observed in *Fmr1* KO mice. Figure 3 shows the average spectral power of 8 mg/kg JZL-184-treated *Fmr1* KO (4 h, *N* = 13; 1 day, *N* = 8) mice 4 h and 1 day post-treatment as a ratio of *Fmr1* KO mice prior to treatment (PRE, *N* = 22). Supplementary Figure S2.1 (Additional file 1) shows the average spectral power of 8 mg/kg JZL-184-treated *Fmr1* KO compared to WT controls.

Statistical analysis for spectral power 4 h and 1 day post-treatment using a two-way ANOVA for each of the six regions separately revealed a significant main effect of treatment for all regions (LF: treatment, *p* = 0.03; RF: treatment, *p* = 0.0014; RM: treatment, *p* = 0.0005; RT: treatment, *p* < 0.0001) except LM and LT (LM: treatment, *p* = 0.39, LT: treatment, *p* = 0.05). There was no significant main effect of frequency or interaction for all regions (see Supplementary Table S3 for summary of statistical results, Additional file 1). Results show a decrease in low gamma power in the regions of the right hemisphere for the 8 mg/kg JZL-184-treated *Fmr1* KO mice (*N* = 13) 4 h post-treatment compared to *Fmr1* KO mice (PRE, *N* = 22) prior to treatment ([RF]: KO PRE, 1 ± 0.11 (mean ± SEM); JZL-184-treated *Fmr1* KO, 0.61 ± 0.09, *p* = 0.32; [RM]: KO PRE, 1 ± 0.11; JZL-184-treated *Fmr1* KO, 0.59 ± 0.10, *p* = 0.27; [RT]: KO PRE, 1 ± 0.15; JZL-184-treated *Fmr1* KO, 0.50 ± 0.08, *p* = 0.17), but post hoc comparisons reveal the reduction was not significant (Fig. 3). Statistical analysis for spectral power 1 day post-treatment revealed no significant changes in baseline spectral power between 8 mg/kg JZL-184-treated *Fmr1* KO and *Fmr1* KO mice prior to treatment (Fig. 3).

In WT mice, statistical analysis for spectral power 4 h and 1 day post-JZL-184 treatment using a two-way ANOVA for each of the six regions separately revealed a significant main effect of treatment for all regions (Supplementary Figure S2.2, LF: treatment, *p* = 0.003; RF: treatment, *p* < 0.0001; LM: treatment, *p* < 0.0001; RM: treatment, *p* < 0.0001; LT: treatment, *p* < 0.0001; RT: treatment, *p* < 0.0001). There was no significant main effect of frequency (LF: *p* = 0.71 (F); RF: *p* = 0.71; LM: *p* = 0.98; RM: *p* = 0.98; LT: *p* = 0.73; RT: *p* = 0.78) or interaction (all regions, *p* = 0.99) for all regions. Post hoc comparisons show that treatment with 8 mg/kg JZL-184 (4 h, *N* = 11) significantly reduced delta power in the LT (*p* = 0.01) region at 4 h post-treatment compared to WT PRE mice (*N* = 24, Supplementary Figure S2.2, Additional File 1). Post hoc comparisons of vehicle-treated *Fmr1* KO mice or vehicle-treated WT compared to controls at 4 h or 1 day post-treatment revealed no significant power spectral density changes (Supplementary Figures S2.3-S2.4, respectively; Additional file 1).

Overall, assessment of resting baseline shows that 4 h post-JZL-184 administration there is a slight reduction in gamma power in the right hemisphere of *Fmr1* KO mice but not 1 day post-treatment. Furthermore, there is a significant reduction in delta power in the left temporal region of WT mice at 4 h but not 1 day post-treatment.

### JZL-184 treatment improves synchronization to auditory stimuli in *Fmr1* KO mice

**Fig. 4:**
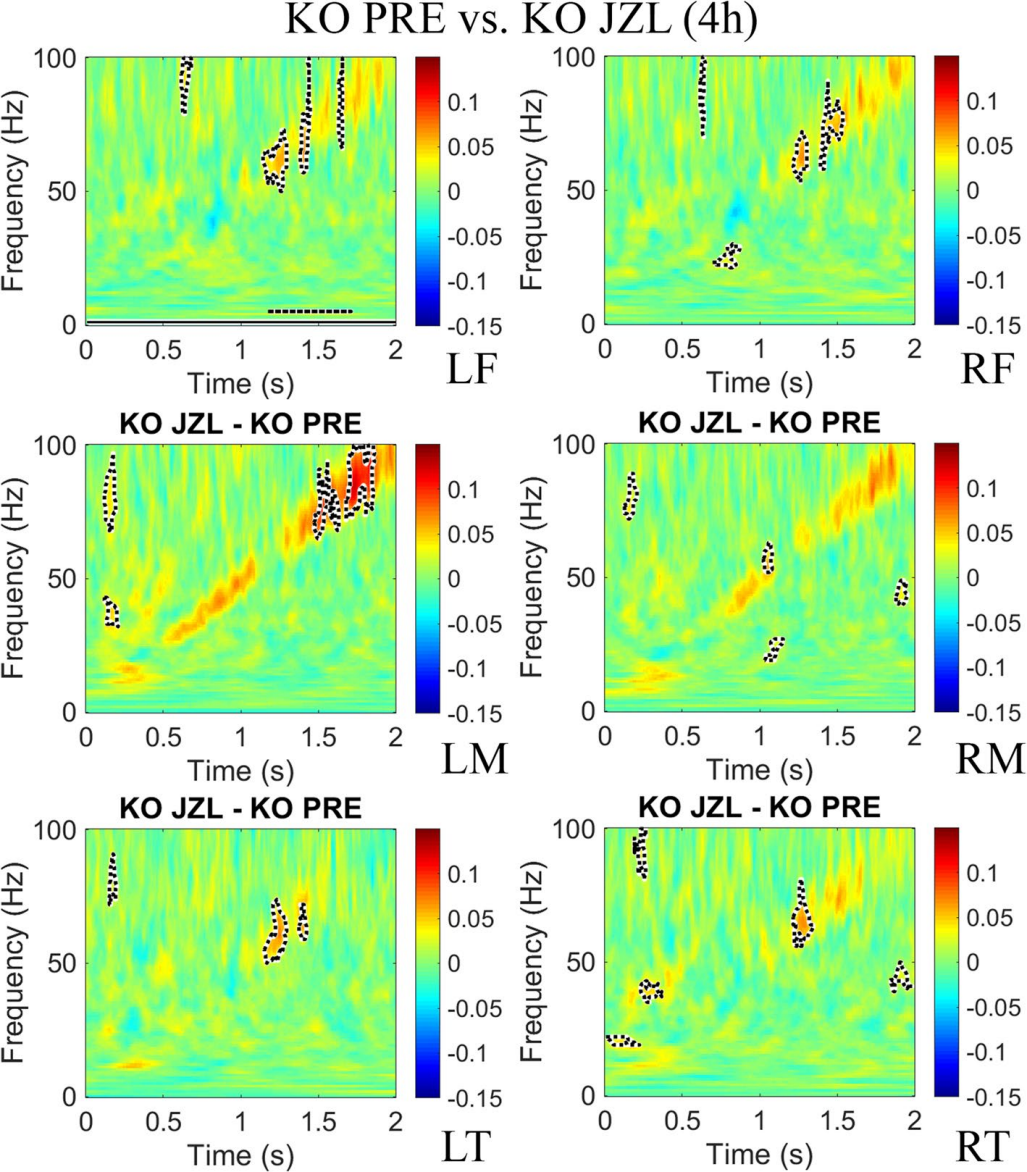
JZL-184 treatment improves inter-trial phase coherence to auditory chirp in *Fmr1* KO mice 4 h post-treatment. Graphs show group mean differences between JZL-184-treated *Fmr1* KO mice (*N* = 13) 4 h post-treatment compared with *Fmr1* KO mice prior to treatment (*N* = 22) in left/right frontal, medial, and temporal regions of the mouse cortex. Monte Carlo statistical method on cluster analysis revealed a significant increase (*p* < 0.025) in average ITPC values in JZL-184-treated *Fmr1* KO mice (yellow and red regions with dotted black outline) in the gamma-band range (~ 30–100 Hz) in all regions. Abbreviations: LF, left frontal; RF, right frontal; LM, left medial; RM, right medial; LT, left temporal; RT, right temporal

**Fig. 5:**
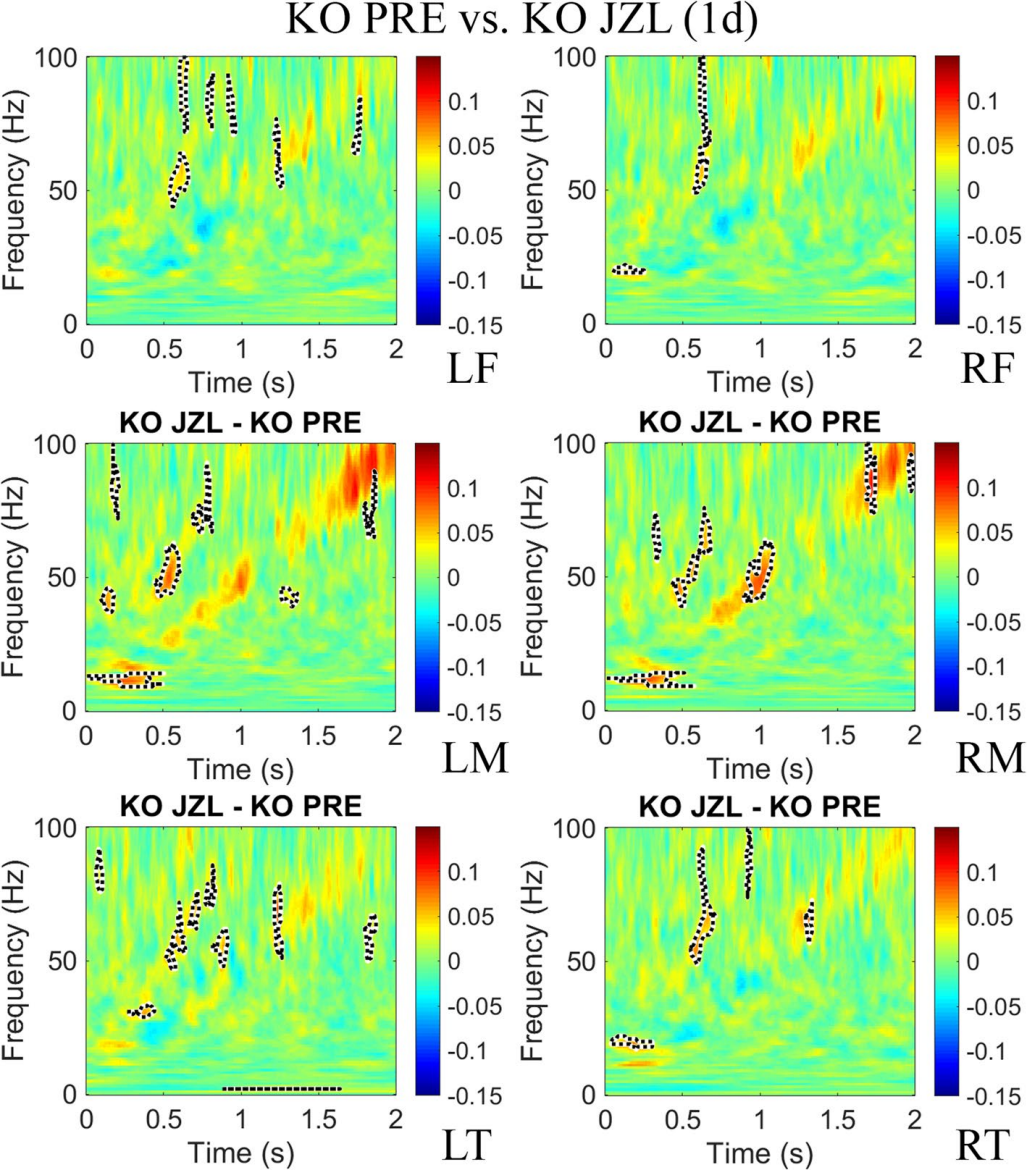
JZL-184 treatment improves inter-trial phase coherence to auditory chirp in *Fmr1* KO mice 1 day post-treatment. Graphs show group mean differences between JZL-184-treated *Fmr1* KO (*N* = 8) mice 1 day post-treatment compared to *Fmr1* KO mice prior to treatment (*N* = 22) in left/right frontal, medial, and temporal regions of the mouse cortex. Monte Carlo statistical method on cluster analysis revealed a significant increase (*p* < 0.025) in ITPC to chirp 1 day post-treatment in the gamma-band range in LF, RM, LT, and RT regions. Abbreviations: LF, left frontal; RF, right frontal; LM, left medial; RM, right medial; LT, left temporal; RT, right temporal

To test whether increasing levels of 2-AG would ameliorate the inter-trial phase coherence (ITPC) deficit to auditory chirp stimuli seen in previous studies with adult *Fmr1* KO mice [28], and humans with FXS [23], MEA responses to repeated chirp stimuli (300 trials) were recorded prior to treatment (PRE), 4 h and 1 day post-treatment. ITPC was calculated across trials in the time × frequency domain using Morlet wavelet analysis [28]. After grand average ITPC was calculated for each group, we found EEG oscillations that matched the frequency of the chirp and were seen as increased ITPC along a diagonal, from 0 to 2 s and 1–100 Hz (ITPC deficits observed in *Fmr1* KO as a ratio of WT mice prior to treatment is shown in Supplementary Figure S4.1, Additional file 1). The means for *Fmr1* KO PRE (*N* = 22) were subtracted from 8 mg/kg JZL-184-treated *Fmr1* KO (*N* = 13) mice 4 h and 1 day post-treatment to show the ITPC difference between the treatment groups (Figs. 4 and 5, respectively) in each of the six regions. Supplementary Figure S4.2 (Additional file 1) shows the ITPC difference of 8 mg/kg JZL-184-treated *Fmr1* KO compared to WT controls. For statistical analysis, clusters of *p*-values were calculated, and significant differences (*p* < 0.025) were overlaid on the chirp response to demonstrate quantitative differences between each group after correction for multiple comparisons [48]. Monte Carlo statistical method on cluster analysis revealed a significant increase in average ITPC values in JZL-184-treated *Fmr1* KO mice in the gamma-band range (~ 30–100 Hz) in all regions (Fig. 4). One day post-treatment, statistical analysis revealed a significant increase in ITPC to chirp in the gamma-band range in LF, RM, LT, and RT regions (Fig. 5).

Interestingly, statistical analysis of average ITPC values in JZL-184-treated WT mice compared to WT PRE mice revealed no significant changes in ITPC to auditory chirp stimuli at 4 h or 1 day post-treatment (Supplementary Figure S4.3, Additional file 1). Vehicle-treated *Fmr1* KO and vehicle-treated WT mice compared to PRE controls revealed a significant reduction in ITPC in the low gamma-band range in the LT region (*Fmr1* KO vehicle vs. KO PRE) and RT (WT vehicle vs. WT PRE) region 4 h post-treatment (Supplementary Figures S4.4-S4.5, respectively). All other regions showed no significant differences in ITPC to auditory chirp stimuli at 4 h or 1 day post-treatment.

These results indicate that acute treatment of the MAGL inhibitor, JZL-184, improves the fidelity of temporal responses to auditory stimuli, specifically in *Fmr1* KO mice during the time window that 2-AG levels are increased (4 h post-treatment) and when 2-AG levels have returned to baseline levels (1 day post-treatment) by increasing gamma-band (~ 30–100 Hz) ITPC in the *Fmr1* KO mouse cortex.

### JZL-184 treatment ameliorates anxiety and hyperactivity in *Fmr1* KO mice

**Fig. 6:**
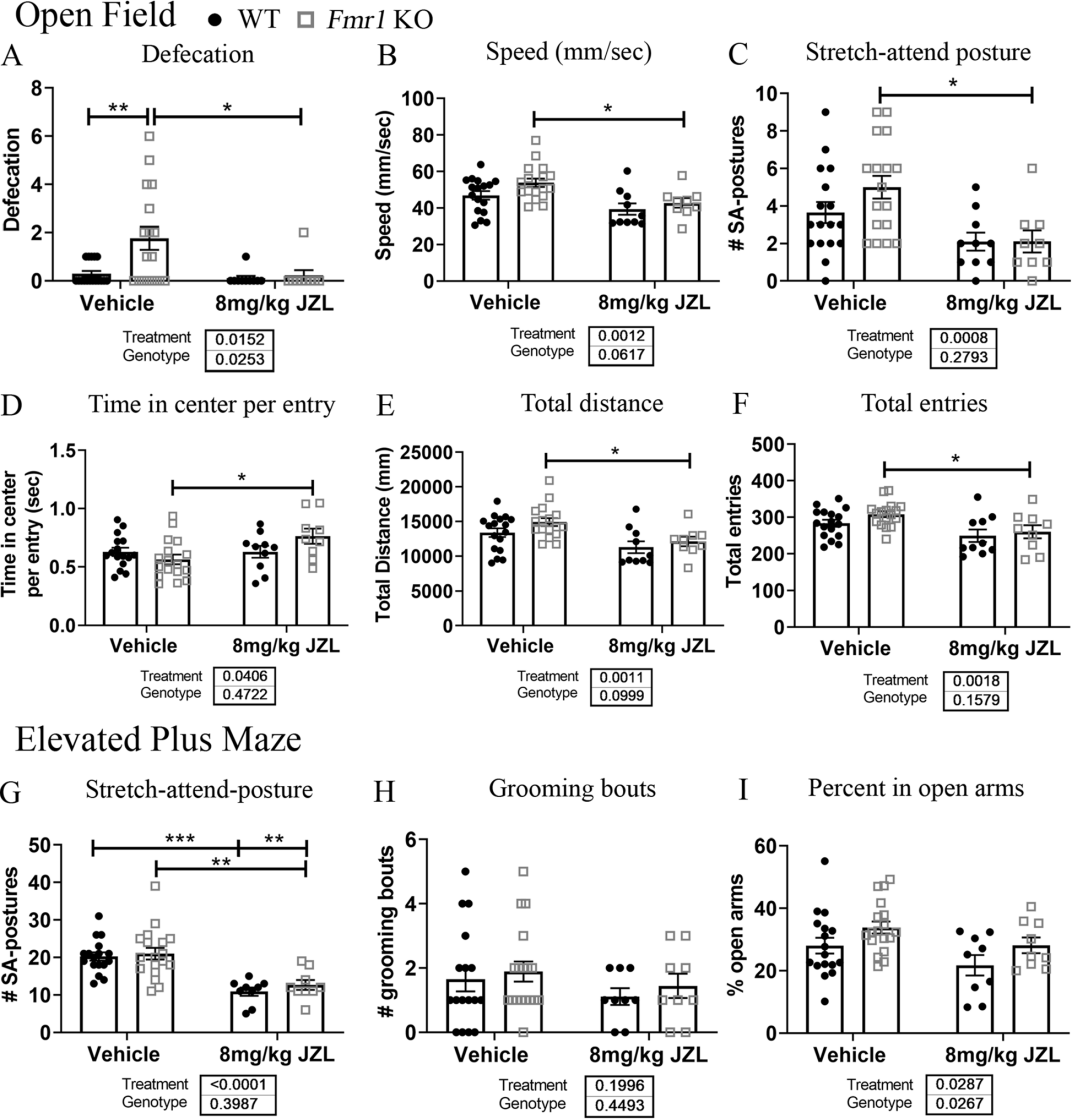
JZL-184 treatment improves anxiety/hyperactivity phenotype associated with *Fmr1* KO mice. Graphs show behavioral measures assessed for anxiety-like behaviors and hyperactivity in the open field and elevated plus maze 4 h post-treatment in *Fmr1* KO and WT mice. Behavioral measures assessed in the open field included the amount of defecation (**A**), speed (mm/s) (**B**), number of stretch-attend (SA) postures (**C**), time in the center per entry (**D**), total distance (**E**), and total entries. **G**–**I** Graphs show EPM behaviors assessed, including the number of stretch-attend postures (**G**), grooming bouts (**H**), and percent time spent in open arms (**I**). Statistical analysis was performed using the two-way ANOVA for all comparisons. Values represent means per group, and error bars represent the standard error of the mean (SEM). *p < 0.05, **p < 0.01, ***p < 0.001, ****p < 0.0001. WT vehicle, *N* = 17; *Fmr1* KO vehicle, *N* = 17–18; WT 8 mg/kg JZL-184, *N* = 9–10; *Fmr1* KO 8 mg/kg JZL-184, *N* = 9. Excluded: WT vehicle, *N* = 1 (D), *Fmr1* KO vehicle, *N* = 1 (OF); WT 8 mg/kg JZL-184, *N* = 1 (EPM)

Next, we assessed whether JZL-184 treatment reduces anxiety-like behaviors and hyperactivity in *Fmr1* KO mice. Mice received injections of 8 mg/kg JZL-184 or vehicle, and anxiety-like behaviors and hyperactivity were assessed with the open field (OF) and elevated plus maze (EPM) 4 h post-treatment. Statistical analysis using a two-way ANOVA revealed significant main effects of treatment in OF measures assessed, including defecation (*p* = 0.02), speed (*p* = 0.001), number of stretch-attend postures (*p* = 0.0008), time in center per entry (*p* = 0.04), total distance (*p* = 0.001), and total entries (*p* = 0.002; see Supplementary Table S5 for a summary of statistical results, Additional file 1). Post hoc comparisons showed that vehicle-treated *Fmr1* KO mice (*N* = 17) had increased defecation (# of fecal droppings) compared to vehicle-treated WT mice (Fig. 6A, N = 17, *p* = 0.005). Treatment with JZL-184 significantly reduced defecation in *Fmr1* KO mice compared to vehicle-treated *Fmr1* KO mice (*p* = 0.02). Post hoc comparisons also showed that JZL-184-treated *Fmr1* KO (*N* = 9) mice had reduced speed (Fig. 6B, p = 0.04), decreased number of stretch-attend postures (Fig. 6C, p = 0.01), increased time in the center per entry (Fig. 6D, p = 0.03), decreased total distance (Fig. 6E, p = 0.04), and decreased total entries (Fig. 6F, p = 0.04) compared to vehicle-treated *Fmr1* KO mice (*N* = 17; see Supplementary Table S6 for a summary of results, Additional file 1).

In the EPM, statistical analysis using a two-way ANOVA revealed a significant effect of treatment for the number of stretch-attend postures (Fig. 6G, p < 0.0001) and percent time spent in the open arms (Fig. 6i, p = 0.03), but not for the number of grooming bouts (Fig. 6h, p = 0.20). Post hoc comparisons showed that treatment with JZL-184 significantly reduced the number of stretch-attend postures in both WT (*N* = 9, *p* = 0.0003) and *Fmr1* KO (*N* = 9, *p* = 0.0013) mice compared to vehicle-treated control mice (Fig. 6G; WT vehicle, *N* = 17; *Fmr1* KO vehicle, *N* = 17).

Overall, these results indicate that JZL-184 treatment reduced anxiety-like behaviors and hyperactivity in *Fmr1* KO-treated mice 4 h post-treatment.

## Discussion

EEG biomarkers observed in the mouse model of FXS, the *Fmr1* KO mouse, are used to assess the efficacy of eCB system modulation as a potential therapeutic for cortical hyperexcitability in FXS. We tested whether JZL-184 treatment would increase 2-AG levels and normalize abnormal EEG phenotypes. The lipidomic analysis confirmed that treatment with JZL-184 significantly increased 2-AG levels in the mouse cortex 4 h post-treatment. Furthermore, statistical analysis of spectral power following JZL-184 treatment revealed a significant main effect of treatment in all regions except the LM/LT and a tendency to reduce abnormal resting EEG gamma-band power in *Fmr1* KO mice. Treatment with JZL-184 also led to increased fidelity of gamma frequency phase locking to time-varying auditory stimuli in JZL-184-treated *Fmr1* KO mice compared to non-treated *Fmr1* KO mice. Behavioral assessment of anxiety-like behavior and hyperactivity also showed improvements in these measures in *Fmr1* KO mice. Overall, these results indicate that JZL-184 treatment significantly increases 2-AG levels and that enhanced levels of 2-AG may function to improve cortical responses to auditory stimuli and reduce auditory hypersensitivity in FXS.

### Endocannabinoid system dysfunction in FXS

Endocannabinoid system dysfunction is implicated in anxiety-like behavior and hyperactivity [58], social avoidance [59], and cognitive impairments [37, 60] observed in FXS. Yet, the underlying neural and molecular mechanisms remain unclear due to the effects of the eCB system on a broad spectrum of neurotransmitters [61] and neuronal cell types [62]. The eCB system comprises endogenous ligands, including 2-AG and AEA, the enzymes regulating their biosynthesis and degradation, and the G protein-coupled receptors, the cannabinoid subtype 1 receptor (CB1R) and CB2R [63]. 2-AG and AEA are synthesized and released postsynaptically by neurons in an activity-dependent manner and degraded by selective lipases [62, 64]. Monoacylglycerol lipase (MAGL) and alpha/beta hydrolase domain 6 (ABHD6) degrade 2-AG [33, 65, 66], and fatty acid amide hydrolase (FAAH) degrades AEA [67]. These endogenous ligands of presynaptically located CB1Rs act as retrograde signals to inhibit neurotransmitter release, including glutamate and gamma-aminobutyric acid (GABA) release [64, 68, 69]. Regulation of inhibitory and excitatory neurotransmitters allows the eCB system to modulate synapse homeostasis and maintain a proper balance of excitatory and inhibitory activity in neuronal circuits [62]. Notably, evidence suggests that eCB system function varies based on neuroanatomical locations. Thus, region-specific dysregulation of the eCB system may contribute to pathology in specific disorders [38, 62, 70].

Currently, both disruption of mGluR5-mediated production of 2-AG [38, 41–43] and enhanced CB1R activation [68], which are downstream of overactivated mGluR5 [8], are suggested to underlie phenotypic traits observed in FXS [39]. Thus, we tested whether enhancing 2-AG levels would normalize abnormal EEG biomarkers observed in the FXS mouse model following acute treatment with the MAGL inhibitor JZL-184. Our lipidomic analysis indicates that JZL-184 treatment increases 2-AG levels in both WT and *Fmr1* KO mice as expected, given previous studies using JZL-184 [31, 43].

Interestingly, treatment with JZL-184 induced changes in AEA and other lipids like OEA, which is structurally related to AEA. Unlike AEA, OEA lacks affinity for CB1R/CB2Rs. But similarly to AEA, it binds to the peroxisome proliferator-activated receptor alpha (PPAR-α) and the G protein-coupled receptor 119 (GPR119) [71]. Our lipidomic results show that 8 mg/kg JZL-184 significantly enhanced levels of AEA, OEA, and DHEA in *Fmr1* KO mice. JZL-184-induced increases in eCB ligands may serve as a promising therapeutic for other neurodevelopmental disorders. For example, studies in children with ASD report lower serum levels of AEA, OEA, and N-palmitoylethanolamine (PEA) suggesting possible hypo-function of the endocannabinoid system [72, 73]. This study also reported no difference in 2-AG levels [72]. Thus, the EEG improvements observed in our study may also be attributed to increased levels of AEA and other lipids. This is supported by other studies targeting AEA modulation and showing significant behavioral improvements in *Fmr1* KO mice [59, 74].

### The role of the endocannabinoid system in behaviors

An increasing number of studies explore the use of exogenous cannabinoids, namely, tetrahydrocannabinol (THC) and cannabidiol (CBD), in various disorders [62, 75–81], including FXS and ASD [36, 58, 82–85]. Cannabis and targeted eCB system modulation ameliorate symptoms associated with FXS and ASD, including cognitive deficits [37, 38], anxiety and hyperactivity [36, 39, 82], sociability [59, 74], restlessness [36], nociception [40, 86], and audiogenic seizure susceptibility [39]. However, there is limited knowledge on the neurophysiological effects induced by cannabinoids and the specific mechanisms of how cannabinoids ameliorate FXS symptomatology. Targeted modulation of the eCB system has emerged as an attractive therapeutic approach for its ability to avoid the deleterious effects of cannabinoids and to help dissect out the key players contributing to specific symptomatology. In our study, we found that JZL-184 treatment significantly reduced anxiety-like behavior and hyperactivity in *Fmr1* KO mice (Fig. 6). It is important to highlight the large variability that exists in reported behavioral results for the *Fmr1* KO mice. In fact, contrasting findings have been observed in various tasks including but not limited to locomotor activity and social behavior [58, 87, 88]. In our results, we only observed a significant genotype effect for defecation, but we did see significant improvements in hyperactivity and anxiety-like measures in *Fmr1* KO mice compared to vehicle-treated *Fmr1* KO controls. This is in line with previous studies also showing that JZL-184 reduces anxiety and hyperactivity [43]. One potential limitation of MAGL inhibition is CB1R desensitization and downregulation which occurs following chronic (six consecutive days) treatment of JZL-184 in specific brain regions [89]. These effects are not observed with FAAH inhibition [89], and studies suggest that repeated AEA administration is less likely to produce adaptive changes at CB1Rs [90]. Thus, ligand diversification may be critical for targeted eCB system modulation of function [89]. As a result, a systematic comparison of a dual combination treatment targeting 2-AG and AEA, like JZL-195 [91] or the use of ABHD6 inhibitors, which also degrade 2-AG [66] may be worthwhile to explore to understand the contribution of eCB system signaling in FXS phenotypes. Overall, studies support a beneficial role for eCB system modulation, but additional research will need to clarify optimal treatment parameters and potential treatment combinations.

### Endocannabinoid system modulation effects on cortical responses

Loss of FMRP results in altered synaptic function and plasticity of both interneurons and pyramidal cells, leading to an imbalance of excitatory and inhibitory neurotransmission [92–95]. Altered function of fast-spiking parvalbumin (PV) interneurons, which are significantly reduced and exhibit delayed development in the auditory cortex [96, 97] of *Fmr1* KO mice, may contribute to abnormal network synchrony and alterations in gamma oscillations [98, 99]. Increased gamma-band power but reduced temporally synchronous and spatially focused neural activity may underlie sensory processing deficits in FXS [23]. Cholecystokinin (CCK) interneurons may also be involved in regulating network activity patterns by regulating the spike timing of subgroups of cell ensembles [100, 101]. Furthermore, a large percentage of CCK interneurons express CB1Rs [102]. Thus, modulation of inhibitory cell populations mediated by eCB system modulation may yield improvements in neural synchronization deficits.

Notably, cannabinoids and CB1R agonists disrupt gamma-band oscillations in humans [103] and in animal studies [104–106]. Specifically, evidence suggests that they decrease the power of neural oscillations, particularly in the gamma and theta bands [107]. It has been suggested that one of the mechanisms by which cannabinoids induce psychosis is by disrupting synchronized neural oscillations [107]. In our study, assessment of spectral power following JZL-184 treatment in *Fmr1* KO mice revealed a significant main effect of treatment in all regions except the LM/LT and a reduction in low gamma-band power in the right hemisphere (Fig. 3). Further assessment of the resting baseline period revealed alterations in power coupling between theta-low gamma and alpha-low gamma using a Pearson’s correlation to assess the relationship between power across different frequencies and/or regions [30, 108]. JZL-184 treatment significantly altered theta-low gamma coupling in JZL-184-treated mice 4 h post-treatment (Supplementary Table S4A, Additional file 1) and alpha-low gamma coupling 1 day post-treatment (Supplementary Table S4B, Additional file 1) compared to non-treated *Fmr1* KO mice. Interestingly, the effects of JZL-184 treatment on spectral power and power coupling in the *Fmr1* KO mice were associated with improvements in cortical responses to auditory stimuli and behavior.

Chirp ITPC has been shown to be a key EEG biomarker of FXS in both humans [25] and mice [27]. Therefore, it is interesting that treatment with JZL-184 selectively improved chirp ITPC in *Fmr1* KO mice and that these effects lasted up to 1 day post-treatment (Figs. 4 and 5). This suggests that the effects of JZL-184 treatment may persist beyond the time window of increased 2-AG levels (Fig. 1). It also suggests that JZL-184 treatment may selectively modulate neural circuits in *Fmr1* KO mice to improve cortical responses to auditory stimuli. A similar effect was observed in a study conducted by Lysenko et al., where treatment with JZL-184 selectively attenuated spontaneous locomotor activity and improved long-term potentiation and long-term memory deficits in a genetic mouse model of Down syndrome [109].

## Conclusions

In conclusion, results show that treatment with JZL-184 significantly increases 2-AG levels in the mouse cortex and improves synchronized cortical responses to auditory chirp stimuli (chirp ITPC). Thus, JZL-184 may serve as a potential therapeutic for FXS by potentially modulating cortical circuits that underlie neural oscillatory patterns and cortical responses to auditory stimuli. JZL-184 treatment also improves hyperactivity and anxiety-like behaviors associated with FXS. Furthermore, our study provides proof-of-principle for concurrent use of brain eCB lipidomic measurements to interpret the effects of eCB modulatory drugs at specific doses and time points. Together, these findings implicate the eCB system as an emerging candidate to normalize neurophysiological features in FXS and suggest that targeted modulation of eCB system signaling may serve a promising treatment approach.

## Supplementary Information

Below is the link to the electronic supplementary material.

Supplementary file1 (DOCX 7384 kb)

## Data Availability

The datasets supporting the conclusions of this article are included within the article and its additional files.

## Abbreviations

2-AG: 2-Arachidonoyl-sn-glycerol
2-OG: 2-Oleoylglycerol
2-LG: 2-Linoleoyl glycerol
ABHD6: Alpha/beta hydrolase domain 6
AC: Auditory cortex
AEA: Anandamide
ASD: Autism spectrum disorder
CBD: Cannabidiol
CCK: Cholecystokinin-expressing interneurons
DAGL: Diacylglycerol lipase
DHAG: 2-Docosahexaenoylglycerol
DHEA: Docosahexaenoyl ethanolamide
eCB: Endocannabinoid
EEG: Electroencephalography
EPM: Elevated plus maze
ERP: Event-related potential
FAAH: Fatty acid amide hydrolase
FFT: Fast Fourier transform
Fmr1 KO: Fragile X mental retardation 1 Gene knock-out
FXS: Fragile X syndrome
FC: Frontal cortex
ITPC: Inter-trial phase coherence
LF: Left frontal
LM: Left medial
LT: Left temporal
MAGL: Monoacylglycerol lipase
OEA: Oleoylethanolamide
OF: Open field
PV: Parvalbumin
RF: Right frontal
RM: Right medial
RT: Right temporal
RRID: Research resource identifier
STP: Single-trial power
THC: Tetrahydrocannabinol

## References

[1] Sutcliffe JS, Nelson DL, Zhang F, Pieretti M, Caskey CT, Saxe D (1992). DNA methylation represses FMR-1 transcription in fragile X syndrome. Hum Mol Genet.

[2] Verkerk AJ, Pieretti M, Sutcliffe JS, Fu YH, Kuhl DP, Pizzuti A (1991). Identification of a gene (FMR-1) containing a CGG repeat coincident with a breakpoint cluster region exhibiting length variation in fragile X syndrome. Cell.

[3] Sharma A, Hoeffer CA, Takayasu Y, Miyawaki T, McBride SM, Klann E (2010). Dysregulation of mTOR signaling in fragile X syndrome. J Neurosci.

[4] Irwin SA, Galvez R, Greenough WT (2000). Dendritic spine structural anomalies in fragile-X mental retardation syndrome. Cereb Cortex.

[5] Cruz-Martin A, Crespo M, Portera-Cailliau C (2010). Delayed stabilization of dendritic spines in fragile X mice. J Neurosci.

[6] Osterweil EK, Krueger DD, Reinhold K, Bear MF (2010). Hypersensitivity to mGluR5 and ERK1/2 leads to excessive protein synthesis in the hippocampus of a mouse model of fragile X syndrome. J Neurosci.

[7] Bagni C, Greenough WT (2005). From mRNP trafficking to spine dysmorphogenesis: the roots of fragile X syndrome. Nat Rev Neurosci.

[8] Huber KM (2002). Altered synaptic plasticity in a mouse model of fragile X mental retardation. Pro Natl Acad Sci USA.

[9] Bear MF, Dolen G, Osterweil E, Nagarajan N (2008). Fragile X: translation in action. Neuropsychopharmacology.

[10] Bear MF, Huber KM, Warren ST (2004). The mGluR theory of fragile X mental retardation. Trends Neurosci.

[11] Arroyo ED, Fiole D, Mantri SS, Huang C, Portera-Cailliau C (2019). Dendritic spines in early postnatal fragile X mice are insensitive to novel sensory experience. J Neurosci.

[12] Padmashri R, Reiner BC, Suresh A, Spartz E, Dunaevsky A (2013). Altered structural and functional synaptic plasticity with motor skill learning in a mouse model of fragile X syndrome. J Neurosci.

[13] Hagerman RJ, Rivera SM, Hagerman PJ (2008). The fragile X family of disorders: a model for autism and targeted treatments. Curr Pediatr Rev.

[14] Kaufmann WE, Cortell R, Kau AS, Bukelis I, Tierney E, Gray RM (2004). Autism spectrum disorder in fragile X syndrome: communication, social interaction, and specific behaviors. Am J Med Genet A.

[15] Talisa VB, Boyle L, Crafa D, Kaufmann WE (2014). Autism and anxiety in males with fragile X syndrome: an exploratory analysis of neurobehavioral profiles from a parent survey. Am J Med Genet A.

[16] Cordeiro L, Ballinger E, Hagerman R, Hessl D (2011). Clinical assessment of DSM-IV anxiety disorders in fragile X syndrome: prevalence and characterization. J Neurodev Disord.

[17] Wolff JJ, Hazlett HC, Lightbody AA, Reiss AL, Piven J (2013). Repetitive and self-injurious behaviors: associations with caudate volume in autism and fragile X syndrome. J Neurodev Disord.

[18] Miller LJ, McIntosh DN, McGrath J, Shyu V, Lampe M, Taylor AK (1999). Electrodermal responses to sensory stimuli in individuals with fragile X syndrome: a preliminary report. Am J Med Genet.

[19] Rotschafer S, Razak K (2013). Altered auditory processing in a mouse model of fragile X syndrome. Brain Res.

[20] Ethridge LE, White SP, Mosconi MW, Wang J, Byerly MJ, Sweeney JA. Reduced habituation of auditory evoked potentials indicate cortical hyper-excitability in Fragile X Syndrome. Transl Psychiatry. 2016;6:e787. 10.1038/tp.2016.48 PMC4872406 27093069

[21] Qiu LF, Hao YH, Li QZ, Xiong ZQ (2008). Fragile X syndrome and epilepsy. Neurosci Bull.

[22] Kazdoba TM, Leach PT, Silverman JL, Crawley JN (2014). Modeling fragile X syndrome in the Fmr1 knockout mouse. Intractable Rare Dis Res.

[23] Ethridge LE, White SP, Mosconi MW, Wang J, Pedapati EV, Erickson CA (2017). Neural synchronization deficits linked to cortical hyper-excitability and auditory hypersensitivity in fragile X syndrome. Mol Autism.

[24] Wang J, Ethridge LE, Mosconi MW, White SP, Binder DK, Pedapati EV (2017). A resting EEG study of neocortical hyperexcitability and altered functional connectivity in fragile X syndrome. J Neurodev Disord.

[25] Ethridge LE, De Stefano LA, Schmitt LM, Woodruff NE, Brown KL, Tran M (2019). Auditory EEG biomarkers in fragile X syndrome: clinical relevance. Front Integr Neurosci.

[26] Kozono N, Okamura A, Honda S, Matsumoto M, Mihara T (2020). Gamma power abnormalities in a Fmr1-targeted transgenic rat model of fragile X syndrome. Sci Rep.

[27] Jonak CR, Lovelace JW, Ethell IM, Razak KA, Binder DK. Multielectrode array analysis of EEG biomarkers in a mouse model of Fragile X Syndrome. Neurobiol Dis. 2020;138:104794. 10.1016/j.nbd.2020.104794 PMC9038039 32036032

[28] Lovelace JW, Ethell IM, Binder DK, Razak KA (2018). Translation-relevant EEG phenotypes in a mouse model of Fragile X Syndrome. Neurobiol Dis.

[29] Wen TH, Lovelace JW, Ethell IM, Binder DK, Razak KA (2019). Developmental changes in EEG phenotypes in a mouse model of fragile X syndrome. Neuroscience.

[30] Pirbhoy PS, Rais M, Lovelace JW, Woodard W, Razak KA, Binder DK (2020). Acute pharmacological inhibition of matrix metalloproteinase-9 activity during development restores perineuronal net formation and normalizes auditory processing in Fmr1 KO mice. J Neurochem.

[31] Long JZ, Li W, Booker L, Burston JJ, Kinsey SG, Schlosburg JE (2009). Selective blockade of 2-arachidonoylglycerol hydrolysis produces cannabinoid behavioral effects. Nat Chem Biol.

[32] Long JZ, Nomura DK, Cravatt BF (2009). Characterization of monoacylglycerol lipase inhibition reveals differences in central and peripheral endocannabinoid metabolism. Chem Biol.

[33] Blankman JL, Simon GM, Cravatt BF (2007). A comprehensive profile of brain enzymes that hydrolyze the endocannabinoid 2-arachidonoylglycerol. Chem Biol.

[34] Mead AP, Welborn M (2018). The untold story of the cannabidiol (CBD) revolution. US Neurology.

[35] Chong MS, Wolff K, Wise K, Tanton C, Winstock A, Silber E (2006). Cannabis use in patients with multiple sclerosis. Mult Scler.

[36] Bar-Lev Schleider L, Mechoulam R, Saban N, Meiri G, Novack V (2019). Real life experience of medical cannabis treatment in autism: analysis of safety and efficacy. Sci Rep.

[37] Qin M, Zeidler Z, Moulton K, Krych L, Xia Z, Smith CB (2015). Endocannabinoid-mediated improvement on a test of aversive memory in a mouse model of fragile X syndrome. Behav Brain Res.

[38] Wang W, Cox BM, Jia Y, Le AA, Cox CD, Jung KM (2018). Treating a novel plasticity defect rescues episodic memory in Fragile X model mice. Mol Psychiatry.

[39] Busquets-Garcia A, Gomis-Gonzalez M, Guegan T, Agustin-Pavon C, Pastor A, Mato S (2013). Targeting the endocannabinoid system in the treatment of fragile X syndrome. Nat Med.

[40] Busquets-Garcia A, Puighermanal E, Pastor A, de la Torre R, Maldonado R, Ozaita A (2011). Differential role of anandamide and 2-arachidonoylglycerol in memory and anxiety-like responses. Biol Psychiatry.

[41] Maccarrone M, Rossi S, Bari M, De Chiara V, Rapino C, Musella A (2010). Abnormal mGlu 5 receptor/endocannabinoid coupling in mice lacking FMRP and BC1 RNA. Neuropsychopharmacology.

[42] Jung KM, Astarita G, Zhu C, Wallace M, Mackie K, Piomelli D (2007). A key role for diacylglycerol lipase-alpha in metabotropic glutamate receptor-dependent endocannabinoid mobilization. Mol Pharmacol.

[43] Jung KM, Sepers M, Henstridge CM, Lassalle O, Neuhofer D, Martin H (2012). Uncoupling of the endocannabinoid signalling complex in a mouse model of fragile X syndrome. Nat Commun.

[44] Argueta DA, DiPatrizio NV (2017). Peripheral endocannabinoid signaling controls hyperphagia in western diet-induced obesity. Physiol Behav.

[45] Argueta DA, Perez PA, Makriyannis A, DiPatrizio NV (2019). Cannabinoid CB1 receptors inhibit gut-brain satiation signaling in diet-induced obesity. Front Physiol.

[46] Perez PA, DiPatrizio NV. Impact of maternal western diet-induced obesity on offspring mortality and peripheral endocannabinoid system in mice. PLoS One. 2018;13(10):e0205021. 10.1371/journal.pone.0205021 PMC6166980 30273406

[47] Jonak CR, Lovelace JW, Ethell IM, Razak KA, Binder DK (2018). Reusable multielectrode array technique for electroencephalography in awake freely moving mice. Front Integr Neurosci.

[48] Lovelace JW, Rais M, Palacios AR, Shuai XS, Bishay S, Popa O (2020). Deletion of Fmr1 from forebrain excitatory neurons triggers abnormal cellular, EEG, and behavioral phenotypes in the auditory cortex of a mouse model of fragile X syndrome. Cereb Cortex.

[49] Artieda J, Valencia M, Alegre M, Olaziregi O, Urrestarazu E, Iriarte J (2004). Potentials evoked by chirp-modulated tones: a new technique to evaluate oscillatory activity in the auditory pathway. Clin Neurophysiol.

[50] Perez-Alcazar M, Nicolas MJ, Valencia M, Alegre M, Iriarte J, Artieda J. Chirp-evoked potentials in the awake and anesthetized rat. A procedure to assess changes in cortical oscillatory activity. Exp Neurol. 2008;210(1):144–53. 10.1016/j.expneurol.2007.10.017 18177639

[51] Purcell DW, John SM, Schneider BA, Picton TW (2004). Human temporal auditory acuity as assessed by envelope following responses. J Acoust Soc Am.

[52] Tallon-Baudry C, Bertrand O, Delpuech C, Pernier J (1996). Stimulus specificity of phase-locked and non-phase-locked 40 Hz visual responses in human. J Neurosci.

[53] Radwan B, Dvorak D, Fenton AA (2016). Impaired cognitive discrimination and discoordination of coupled theta-gamma oscillations in Fmr1 knockout mice. Neurobiol Dis.

[54] Maris E, Oostenveld R (2007). Nonparametric statistical testing of EEG- and MEG-data. J Neurosci Methods.

[55] Sidhu H, Dansie LE, Hickmott PW, Ethell DW, Ethell IM (2014). Genetic removal of matrix metalloproteinase 9 rescues the symptoms of fragile X syndrome in a mouse model. J Neurosci.

[56] Yan QJ, Rammal M, Tranfaglia M, Bauchwitz RP (2005). Suppression of two major Fragile X Syndrome mouse model phenotypes by the mGluR5 antagonist MPEP. Neuropharmacology.

[57] Yan QJ, Asafo-Adjei PK, Arnold HM, Brown RE, Bauchwitz RP (2004). A phenotypic and molecular characterization of the fmr1-tm1Cgr fragile X mouse. Genes Brain Behav.

[58] Zieba J, Sinclair D, Sebree T, Bonn-Miller M, Gutterman D, Siegel S (2019). Cannabidiol (CBD) reduces anxiety-related behavior in mice via an FMRP-independent mechanism. Pharmacol Biochem Behav.

[59] Wei D, Dinh D, Lee D, Li D, Anguren A, Moreno-Sanz G (2016). Enhancement of anandamide-mediated endocannabinoid signaling corrects autism-related social impairment. Cannabis Cannabinoid Res.

[60] Gomis-Gonzalez M, Busquets-Garcia A, Matute C, Maldonado R, Mato S, Ozaita A. Possible therapeutic doses of cannabinoid type 1 receptor antagonist reverses key alterations in fragile X syndrome mouse model. Genes (Basel). 2016;7(9). 10.3390/genes7090056 PMC5042387 27589806

[61] Schlicker E, Kathmann M (2001). Modulation of transmitter release via presynaptic cannabinoid receptors. Trends Pharmacol Sci.

[62] Fernandez-Ruiz J, Galve-Roperh I, Sagredo O, Guzman M (2020). Possible therapeutic applications of cannabis in the neuropsychopharmacology field. Eur Neuropsychopharmacol.

[63] Pertwee RG, Howlett AC, Abood ME, Alexander SP, Di Marzo V, Elphick MR, et al. International Union of Basic and Clinical Pharmacology. LXXIX. Cannabinoid receptors and their ligands: beyond CB(1) and CB(2). Pharmacol Rev. 2010;62(4):588–631. 10.1124/pr.110.003004 PMC2993256 21079038

[64] Katona I, Freund TF (2012). Multiple functions of endocannabinoid signaling in the brain. Annu Rev Neurosci.

[65] Dinh TP, Freund TF, Piomelli D (2002). A role for monoglyceride lipase in 2-arachidonoylglycerol inactivation. Chem Phys Lipids.

[66] Marrs WR, Blankman JL, Horne EA, Thomazeau A, Lin YH, Coy J (2010). The serine hydrolase ABHD6 controls the accumulation and efficacy of 2-AG at cannabinoid receptors. Nat Neurosci.

[67] Cravatt BF, Giang DK, Mayfield SP, Boger DL, Lerner RA, Gilula NB (1996). Molecular characterization of an enzyme that degrades neuromodulatory fatty-acid amides. Nature.

[68] Kano M, Ohno-Shosaku T, Hashimotodani Y, Uchigashima M, Watanabe M (2009). Endocannabinoid-mediated control of synaptic transmission. Physiol Rev.

[69] Katona I, Sperlagh B, Sik A, Kafalvi A, Vizi ES, Mackie K (1999). Presynaptically located CB1 cannabinoid receptors regulate GABA release from axon terminals of specific hippocampal interneurons. J Neurosci.

[70] Wang W, Trieu BH, Palmer LC, Jia Y, Pham DT, Jung KM, et al. A primary cortical input to hippocampus expresses a pathway-specific and endocannabinoid-dependent form of long-term potentiation. eNeuro. 2016;3(4). 10.1523/ENEURO.0160-16.2016 PMC4976302 27517090

[71] Brown AJ (2007). Novel cannabinoid receptors. Br J Pharmacol.

[72] Aran A, Eylon M, Harel M, Polianski L, Nemirovski A, Tepper S (2019). Lower circulating endocannabinoid levels in children with autism spectrum disorder. Mol Autism.

[73] Karhson DS, Krasinska KM, Dallaire JA, Libove RA, Phillips JM, Chien AS (2018). Plasma anandamide concentrations are lower in children with autism spectrum disorder. Mol Autism.

[74] Servadio M, Melancia F, Manduca A, di Masi A, Schiavi S, Cartocci V, et al. Targeting anandamide metabolism rescues core and associated autistic-like symptoms in rats prenatally exposed to valproic acid. Transl Psychiatry. 2016;6(9):e902. 10.1038/tp.2016.182 PMC5048215 27676443

[75] Palmieri B, Laurino C, Vadala M (2017). Short-term efficacy of CBD-enriched hemp oil in girls with dysautonomic syndrome after human papillomavirus vaccination. Isr Med Assoc J.

[76] Puighermanal E, Marsicano G, Busquets-Garcia A, Lutz B, Maldonado R, Ozaita A (2009). Cannabinoid modulation of hippocampal long-term memory is mediated by mTOR signaling. Nat Neurosci.

[77] Zuardi AW (2008). Cannabidiol: from an inactive cannabinoid to a drug with wide spectrum of action. Braz J Psychiatry.

[78] Almeida V, Levin R, Peres FF, Niigaki ST, Calzavara MB, Zuardi AW (2013). Cannabidiol exhibits anxiolytic but not antipsychotic property evaluated in the social interaction test. Prog Neuropsychopharmacol Biol Psychiatry.

[79] Bergamaschi MM, Queiroz RH, Chagas MH, de Oliveira DC, De Martinis BS, Kapczinski F (2011). Cannabidiol reduces the anxiety induced by simulated public speaking in treatment-naive social phobia patients. Neuropsychopharmacology.

[80] Leweke FM, Piomelli D, Pahlisch F, Muhl D, Gerth CW, Hoyer C, et al. Cannabidiol enhances anandamide signaling and alleviates psychotic symptoms of schizophrenia. Transl Psychiatry. 2012;2:e94. 10.1038/tp.2012.15 PMC3316151 22832859

[81] Kaplan JS, Stella N, Catterall WA, Westenbroek RE (2017). Cannabidiol attenuates seizures and social deficits in a mouse model of Dravet syndrome. Pro Natl Acad Sci USA.

[82] Heussler H, Cohen J, Silove N, Tich N, Bonn-Miller MO, Du W (2019). A phase 1/2, open-label assessment of the safety, tolerability, and efficacy of transdermal cannabidiol (ZYN002) for the treatment of pediatric fragile X syndrome. J Neurodev Disord.

[83] Barchel D, Stolar O, De-Haan T, Ziv-Baran T, Saban N, Fuchs DO (2018). Oral cannabidiol use in children with autism spectrum disorder to treat related symptoms and co-morbidities. Front Pharmacol.

[84] Poleg S, Golubchik P, Offen D, Weizman A (2019). Cannabidiol as a suggested candidate for treatment of autism spectrum disorder. Prog Neuropsychopharmacol Biol Psychiatry.

[85] Tartaglia N, Bonn-Miller M, Hagerman R (2019). Treatment of fragile X syndrome with cannabidiol: a case series study and brief review of the literature. Cannabis Cannabinoid Res.

[86] Ramirez-Lopez A, Pastor A, de la Torre R, La Porta C, Ozaita A, Cabanero D, et al. Role of the endocannabinoid system in a mouse model of Fragile X undergoing neuropathic pain. Eur J Pain. 2021. 10.1002/ejp.1753 33619843

[87] Sinclair D, Oranje B, Razak KA, Siegel SJ, Schmid S (2017). Sensory processing in autism spectrum disorders and Fragile X syndrome-From the clinic to animal models. Neurosci Biobehav Rev.

[88] Sorensen EM, Bertelsen F, Weikop P, Skovborg MM, Banke T, Drasbek KR, et al. Hyperactivity and lack of social discrimination in the adolescent Fmr1 knockout mouse. Behav Pharmacol. 2015;26(8 Spec No):733–40. 10.1097/FBP.0000000000000152 26110222

[89] Schlosburg JE, Blankman JL, Long JZ, Nomura DK, Pan B, Kinsey SG (2010). Chronic monoacylglycerol lipase blockade causes functional antagonism of the endocannabinoid system. Nat Neurosci.

[90] Falenski KW, Thorpe AJ, Schlosburg JE, Cravatt BF, Abdullah RA, Smith TH (2010). FAAH-/- mice display differential tolerance, dependence, and cannabinoid receptor adaptation after delta 9-tetrahydrocannabinol and anandamide administration. Neuropsychopharmacology.

[91] Long JZ, Nomura DK, Vann RE, Walentiny DM, Booker L, Jin X (2009). Dual blockade of FAAH and MAGL identifies behavioral processes regulated by endocannabinoid crosstalk in vivo. Pro Natl Acad Sci USA.

[92] Santos AR, Kanellopoulos AK, Bagni C (2014). Learning and behavioral deficits associated with the absence of the fragile X mental retardation protein: what a fly and mouse model can teach us. Learn Mem.

[93] Paluszkiewicz SM, Martin BS, Huntsman MM (2011). Fragile X syndrome: the GABAergic system and circuit dysfunction. Dev Neurosci.

[94] Paluszkiewicz SM, Olmos-Serrano JL, Corbin JG, Huntsman MM (2011). Impaired inhibitory control of cortical synchronization in fragile X syndrome. J Neurophysiol.

[95] Gibson JR, Bartley AF, Hays SA, Huber KM (2008). Imbalance of neocortical excitation and inhibition and altered UP states reflect network hyperexcitability in the mouse model of fragile X syndrome. J Neurophysiol.

[96] Wen TH, Afroz S, Reinhard SM, Palacios AR, Tapia K, Binder DK (2018). Genetic reduction of matrix metalloproteinase-9 promotes formation of perineuronal nets around parvalbumin-expressing interneurons and normalizes auditory cortex responses in developing Fmr1 knock-out mice. Cereb Cortex.

[97] Kulinich AO, Reinhard SM, Rais M, Lovelace JW, Scott V, Binder DK, et al. Beneficial effects of sound exposure on auditory cortex development in a mouse model of Fragile X Syndrome. Neurobiol Dis. 2020;134:104622. 10.1016/j.nbd.2019.104622 31698054

[98] Vreugdenhil M, Jefferys JG, Celio MR, Schwaller B (2003). Parvalbumin-deficiency facilitates repetitive IPSCs and gamma oscillations in the hippocampus. J Neurophysiol.

[99] Sohal VS, Zhang F, Yizhar O, Deisseroth K (2009). Parvalbumin neurons and gamma rhythms enhance cortical circuit performance. Nature.

[100] Klausberger T, Marton LF, O'Neill J, Huck JH, Dalezios Y, Fuentealba P (2005). Complementary roles of cholecystokinin- and parvalbumin-expressing GABAergic neurons in hippocampal network oscillations. J Neurosci.

[101] Lee SY, Soltesz I (2011). Cholecystokinin: a multi-functional molecular switch of neuronal circuits. Dev Neurobiol.

[102] Bodor AL, Katona I, Nyiri G, Mackie K, Ledent C, Hajos N (2005). Endocannabinoid signaling in rat somatosensory cortex: laminar differences and involvement of specific interneuron types. J Neurosci.

[103] Cortes-Briones J, Skosnik PD, Mathalon D, Cahill J, Pittman B, Williams A (2015). Delta9-THC disrupts gamma (gamma)-band neural oscillations in humans. Neuropsychopharmacology.

[104] Hajos M, Hoffmann WE, Kocsis B (2008). Activation of cannabinoid-1 receptors disrupts sensory gating and neuronal oscillation: relevance to schizophrenia. Biol Psychiatry.

[105] Morgan NH, Stanford IM, Woodhall GL. Modulation of network oscillatory activity and GABAergic synaptic transmission by CB1 cannabinoid receptors in the rat medial entorhinal cortex. Neural Plast. 2008;2008:808564. 10.1155/2008/808564 PMC2593022 19079598

[106] Robbe D, Montgomery SM, Thome A, Rueda-Orozco PE, McNaughton BL, Buzsaki G (2006). Cannabinoids reveal importance of spike timing coordination in hippocampal function. Nat Neurosci.

[107] Skosnik PD, Cortes-Briones JA, Hajos M (2016). It’s all in the rhythm: the role of cannabinoids in neural oscillations and psychosis. Biol Psychiatry.

[108] Wang J, Barstein J, Ethridge LE, Mosconi MW, Takarae Y, Sweeney JA (2013). Resting state EEG abnormalities in autism spectrum disorders. J Neurodev Disord.

[109] Lysenko LV, Kim J, Henry C, Tyrtyshnaia A, Kohnz RA, Madamba F, et al. Monoacylglycerol lipase inhibitor JZL184 improves behavior and neural properties in Ts65Dn mice, a model of down syndrome. PLoS One. 2014;9(12):e114521. 10.1371/journal.pone.0114521 PMC4256450 25474204

