# Supplementary material for: Increased 2-arachidonoyl-sn-glycerol levels normalize cortical responses to sound and improve behaviors in Fmr1 KO mice
Source: J Neurodev Disord. 2021 Oct 13;13:47. doi: 10.1186/s11689-021-09394-x (PMC8513313; doi:10.1186/s11689-021-09394-x)
Supplement: Supplementary file 1 — Supplementary file1 (DOCX 7384 kb) [file 11689_2021_9394_MOESM1_ESM.docx]

**
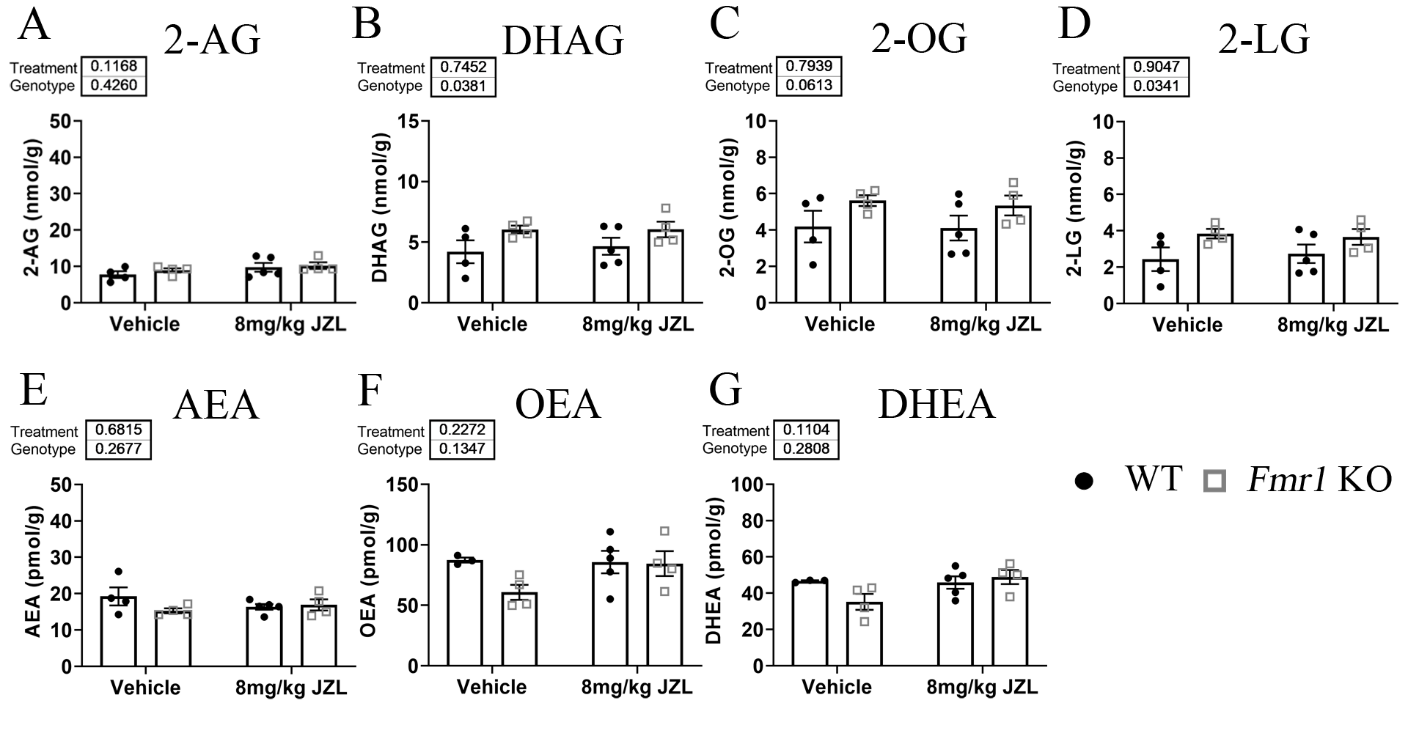
**

**Supplementary Figure S1.** **Effects of JZL-184 treatment on mouse cortical lipidomic profile 1d post-treatment.** Mouse brain levels of 2-arachidonoylglycerol (2-AG, A), docosahexaenoylglycerol (DHAG, B), 2-oleoylglycerol (2-OG, C), 2-linoleoyl glycerol (2-LG, D), anandamide (AEA, E), oleoylethanolamide (OEA, F), and docosahexaenoyl ethanolamide (DHEA, G) 1 day post treatment in *Fmr1* KO and WT mice treated with 8 mg/kg JZL-184 or vehicle. Assessment of lipid levels 1d post treatment using two-way ANOVA revealed no significant changes in vehicle-treated or 8mg/kg JZL-184-treated *Fmr1* KO or WT mice. Values represent means and error bars represent standard error of the mean (SEM). Vehicle WT, N=3-4 per group; 8mg/kg JZL-184 WT, N=5 per group; All *Fmr1* KO groups, N=4 per group. Excluded: OEA/DHEA, vehicle WT, N=1. *p<0.05, **p<0.01, ***p<0.001, ****p<0.0001.


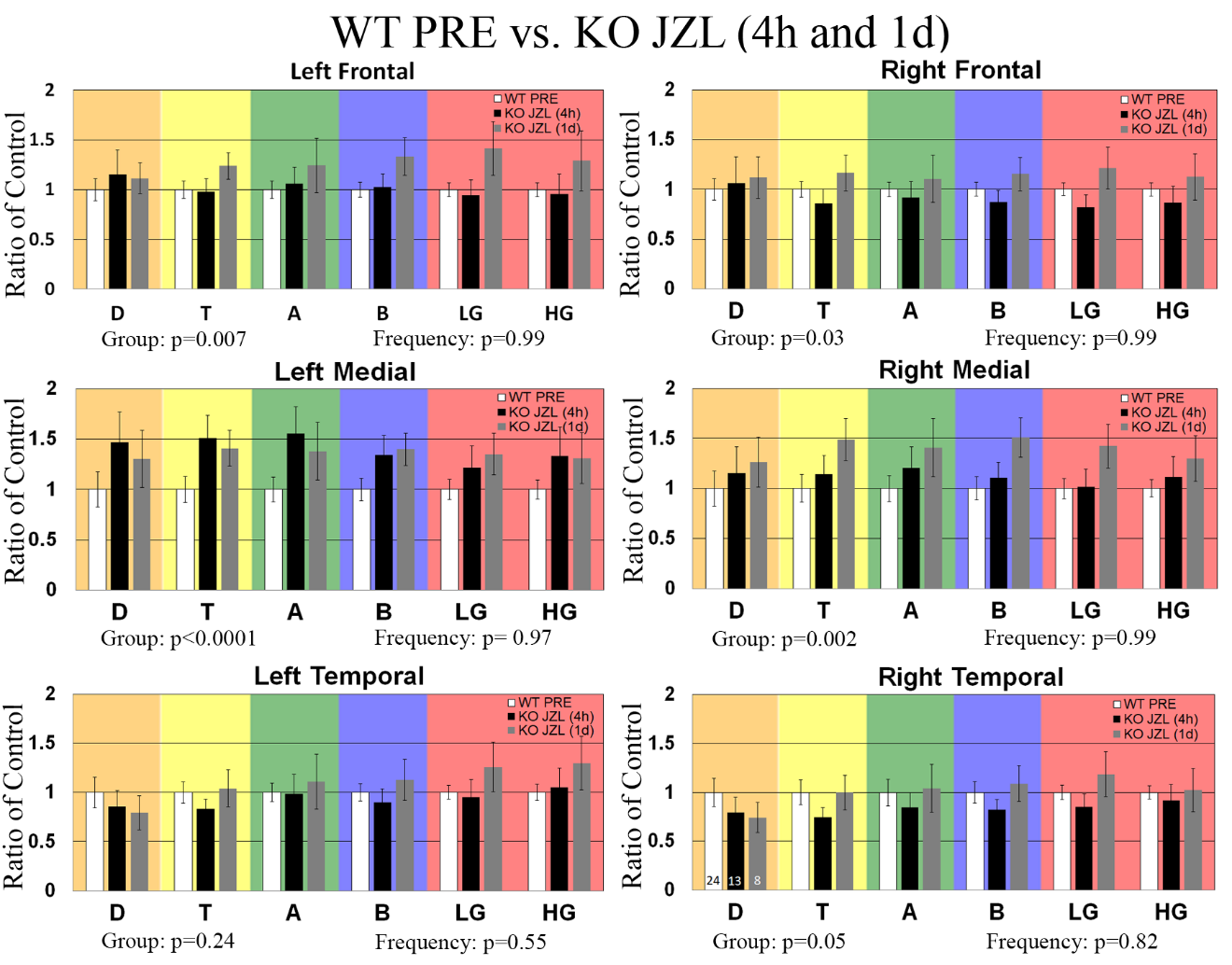


**Supplementary Figure S2.1. Effects of 8mg/kg JZL184 treatment on spectral power in *Fmr1* KO mice compared to WT controls.** Resting baseline was recorded 4 hours (4h) and 1 day (1d) post treatment. Graphs show average spectral power of 8mg/kg JZL184-treated *Fmr1* KO mice (4h, N=13; 1d, N=8) as a ratio of WT mice prior to treatment (PRE, N=24). Statistical analysis was performed using two-way ANOVA for all comparisons. Values represent means per group and error bars represent standard error of the mean (SEM). Abbreviations: D, delta; T, theta; A, alpha; B, beta; LG, low gamma; HG, high gamma.

**
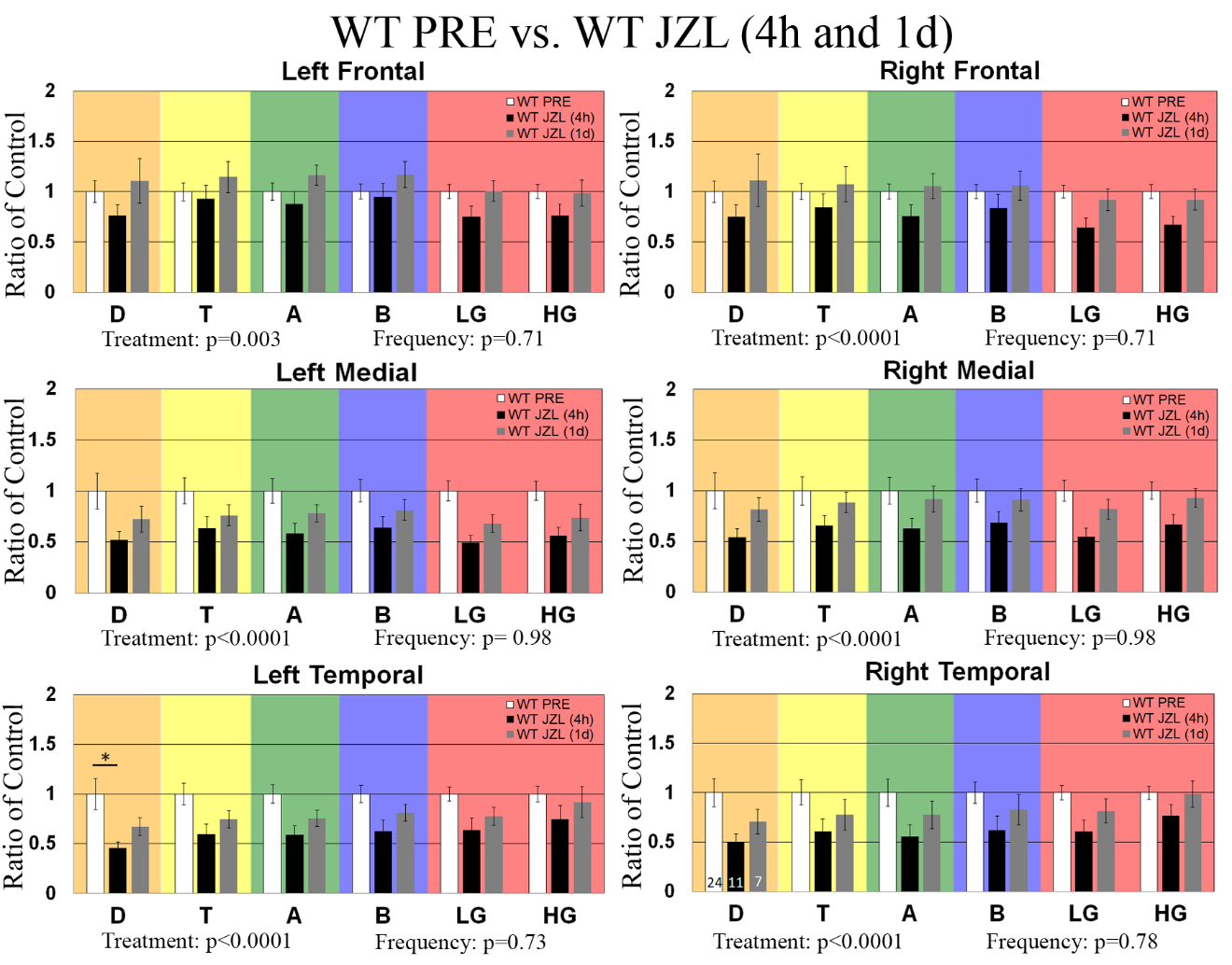
**

**Supplementary Figure S2.2. Effects of 8mg/kg JZL184 treatment on spectral power in WT mice compared to WT controls.** Resting baseline was recorded 4 hours and 1 day post treatment. Graphs show average spectral power of 8mg/kg JZL184-treated WT mice (4h, N=11; 1d, N=7) as a ratio of WT mice prior to treatment (PRE, N=24). Statistical analysis was performed using two-way ANOVA for all comparisons. Values represent means per group and error bars represent standard error of the mean (SEM). *p<0.05, **p<0.01, ***p<0.001, ****p<0.0001. Abbreviations: D, delta; T, theta; A, alpha; B, beta; LG, low gamma; HG, high gamma.


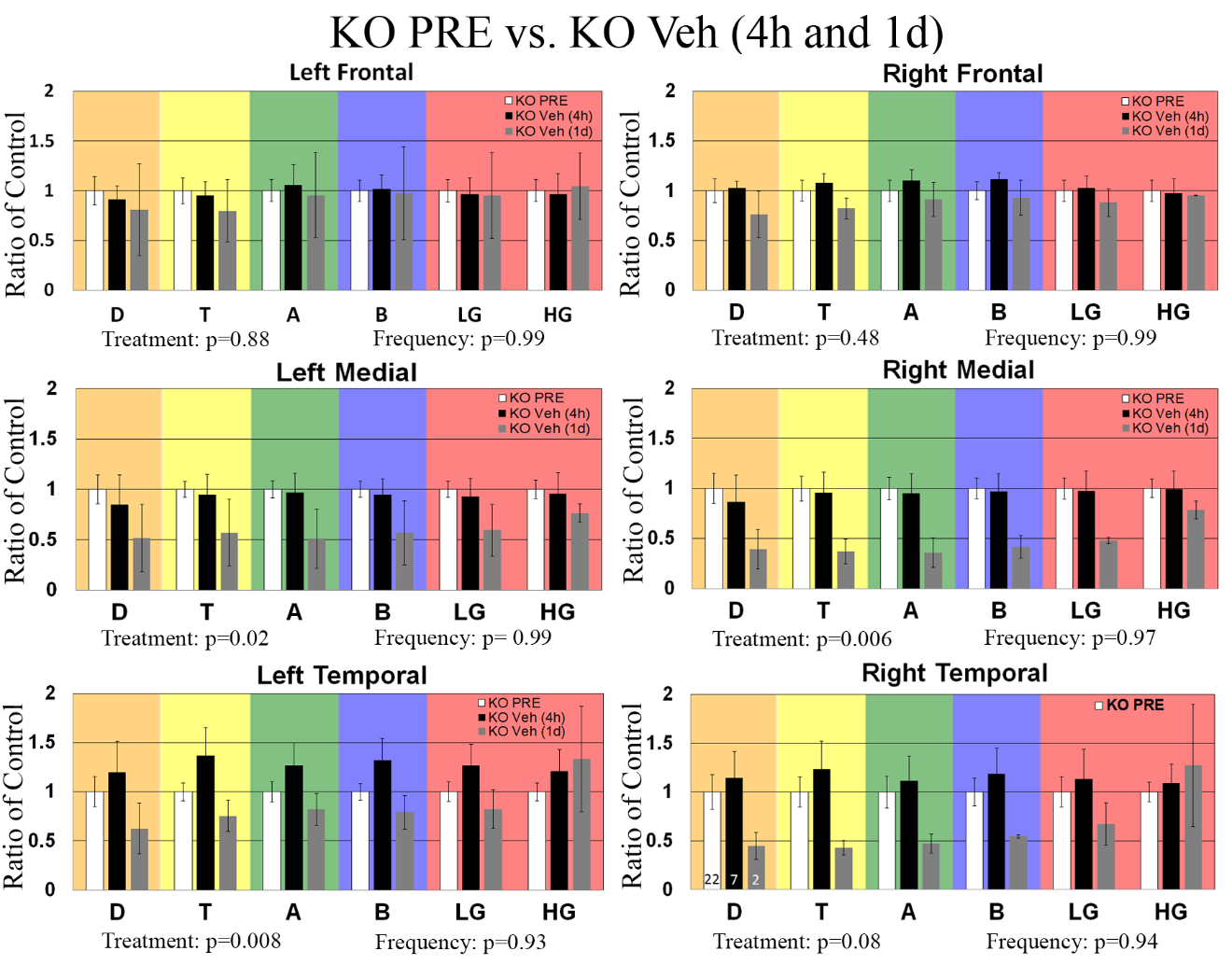


**Supplementary Figure S2.3. Effects of vehicle treatment on spectral power in *Fmr1* KO mice compared to controls.** Resting baseline was recorded 4 hours and 1 day post treatment. Graphs show average spectral power of vehicle treated *Fmr1* KO mice (4h, N=7; 1d, N=2) as a ratio of *Fmr1* KO mice prior to treatment (PRE, N=22). Statistical analysis was performed using two-way ANOVA for all comparisons. Values represent means per group and error bars represent standard error of the mean (SEM). Abbreviations: D, delta; T, theta; A, alpha; B, beta; LG, low gamma; HG, high gamma.


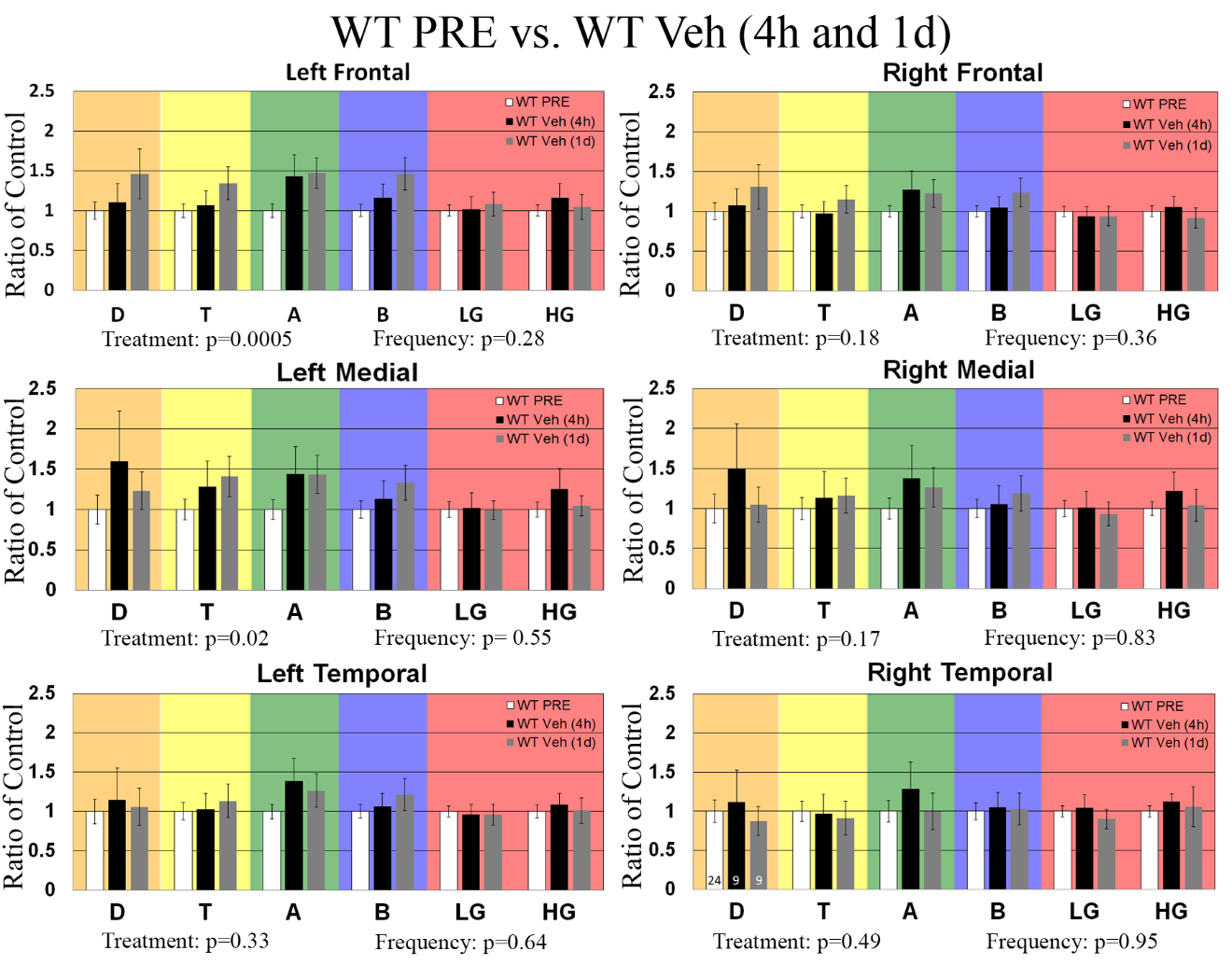


**Supplementary Figure S2.4. Effects of vehicle treatment on spectral power in WT mice compared to controls.** Resting baseline was recorded 4 hours and 1 day post treatment. Graphs show average spectral power of vehicle treated WT mice (4h, N=9; 1d, N=9) as a ratio of WT mice prior to treatment (PRE, N=24). Statistical analysis was performed using two-way ANOVA for all comparisons. Values represent means per group and error bars represent standard error of the mean (SEM). Abbreviations: D, delta; T, theta; A, alpha; B, beta; LG, low gamma; HG, high gamma.


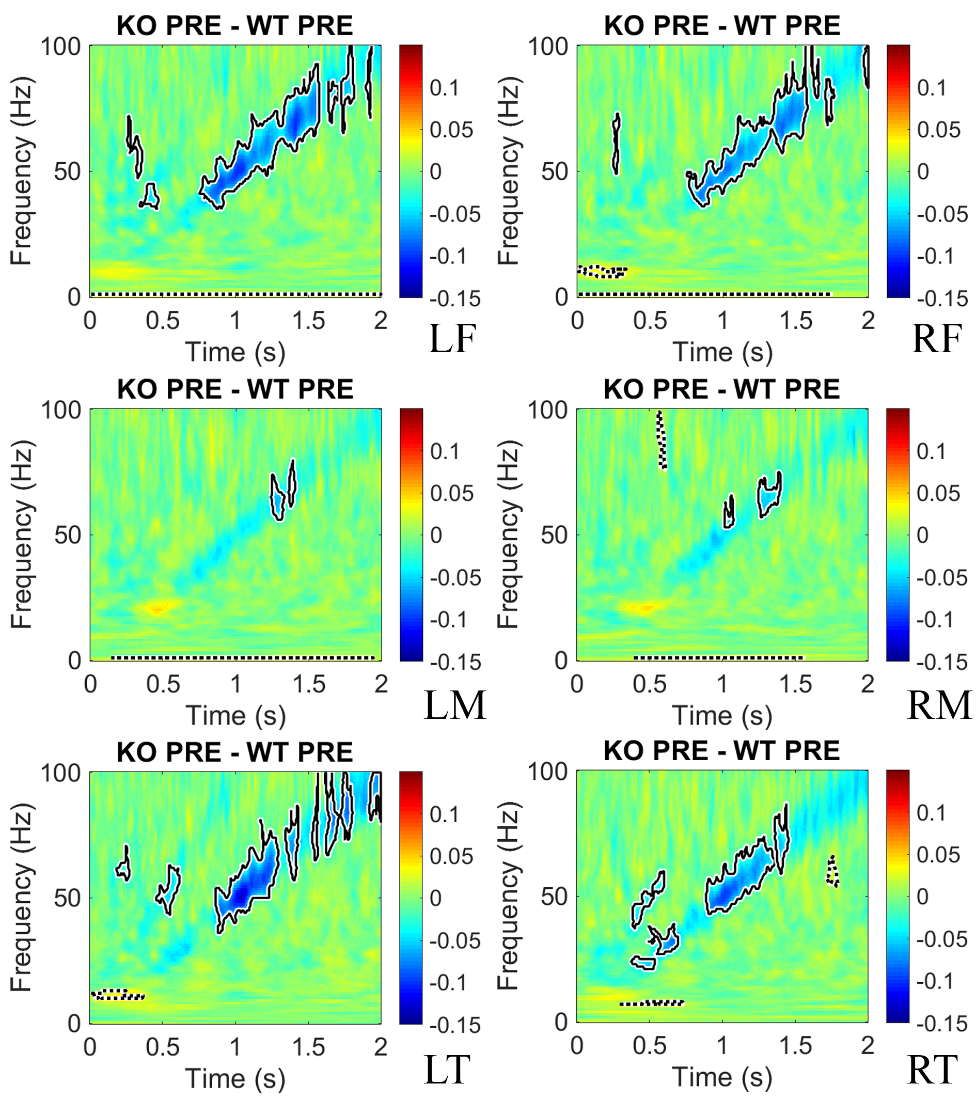


**Supplementary Figure S4.1. Chirp ITPC in *Fmr1* KO and WT mice prior to treatment.** Graphs show ITPC difference between *Fmr1* KO (N=22) mice and WT (N=24) mice prior to treatment. *Fmr1* KO mice exhibit a deficit in ITPC compared to WT mice in all regions. Abbreviations: left/right frontal (LF/RF), left/right medial (LM/RM), left/right temporal (LT/RT).


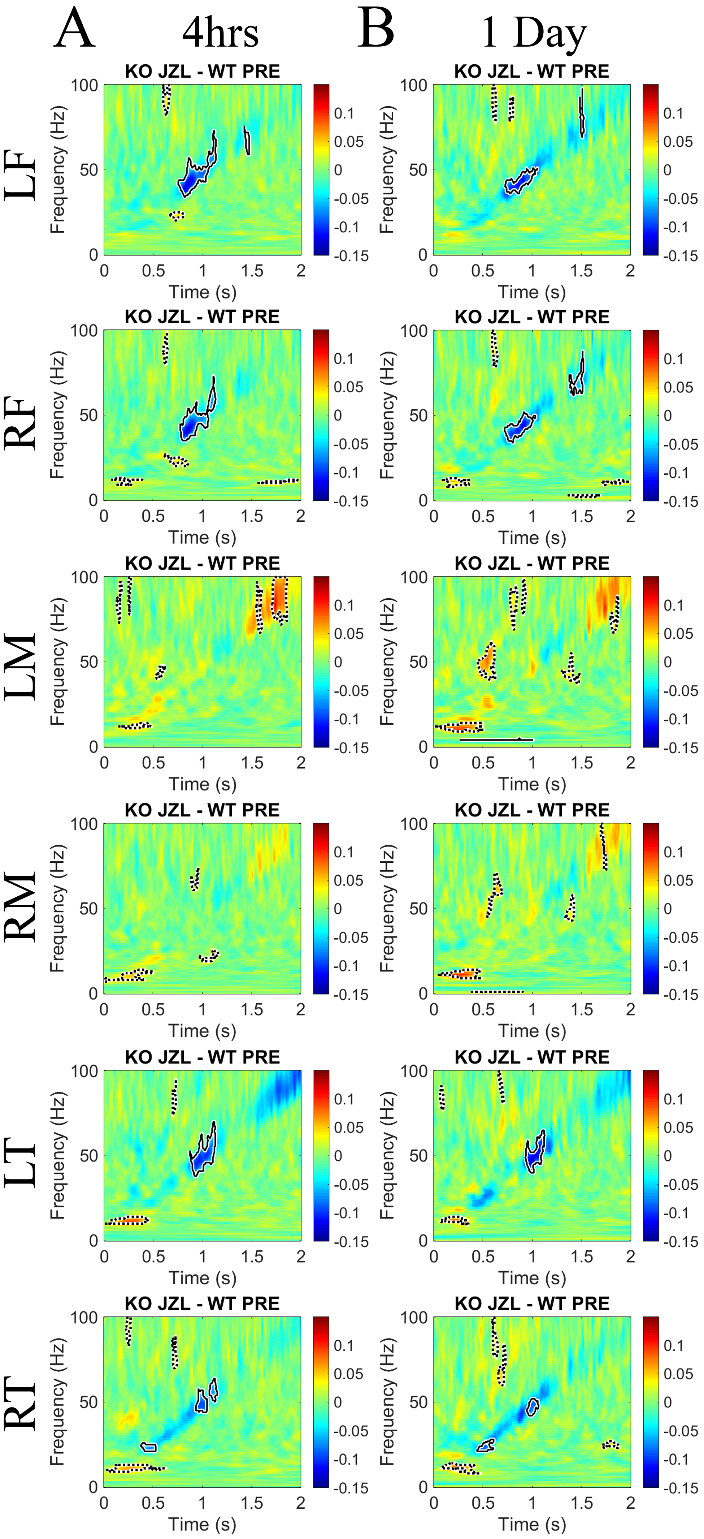


**Supplementary Figure S4.2. Chirp ITPC in 8mg/kg JZL-184 treated *Fmr1* KO mice compared to WT mice.** A-B) Graphs show ITPC difference between 4 hour and 1-day 8mg/kg JZL184 treated *Fmr1* KO mice (4h, N=13; 1d, N=8) and WT mice prior to treatment (PRE, N=24). Abbreviations: left/right frontal (LF/RF), left/right medial (LM/RM), left/right temporal (LT/RT).

**
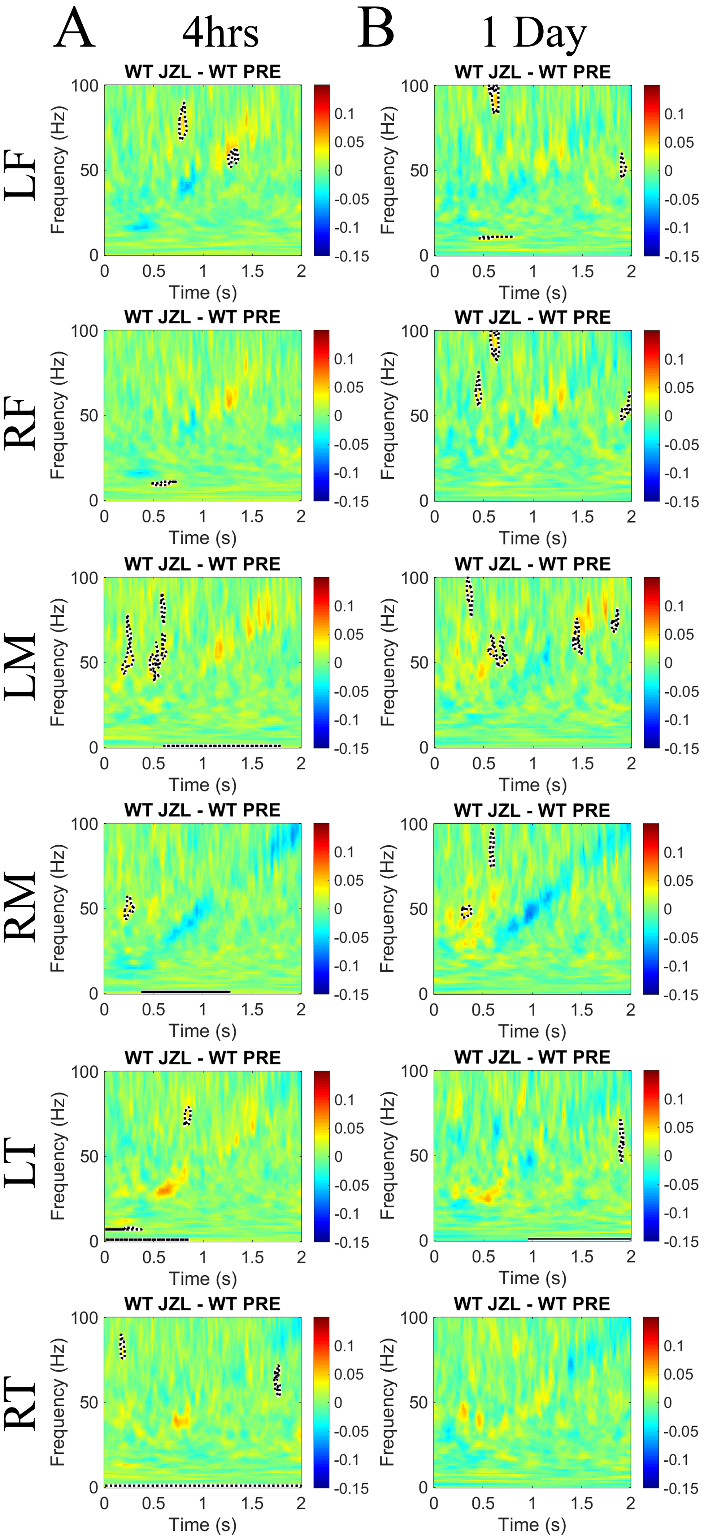
**

**Supplementary Figure S4.3. Chirp ITPC in 8mg/kg JZL-184 treated WT mice compared to controls.** A-B) Graphs show ITPC difference between 4 hour and 1-day 8mg/kg JZL184 treated WT mice (4h, N=11; 1d, N=7) and WT mice prior to treatment (PRE, N=24). Abbreviations: left/right frontal (LF/RF), left/right medial (LM/RM), left/right temporal (LT/RT).


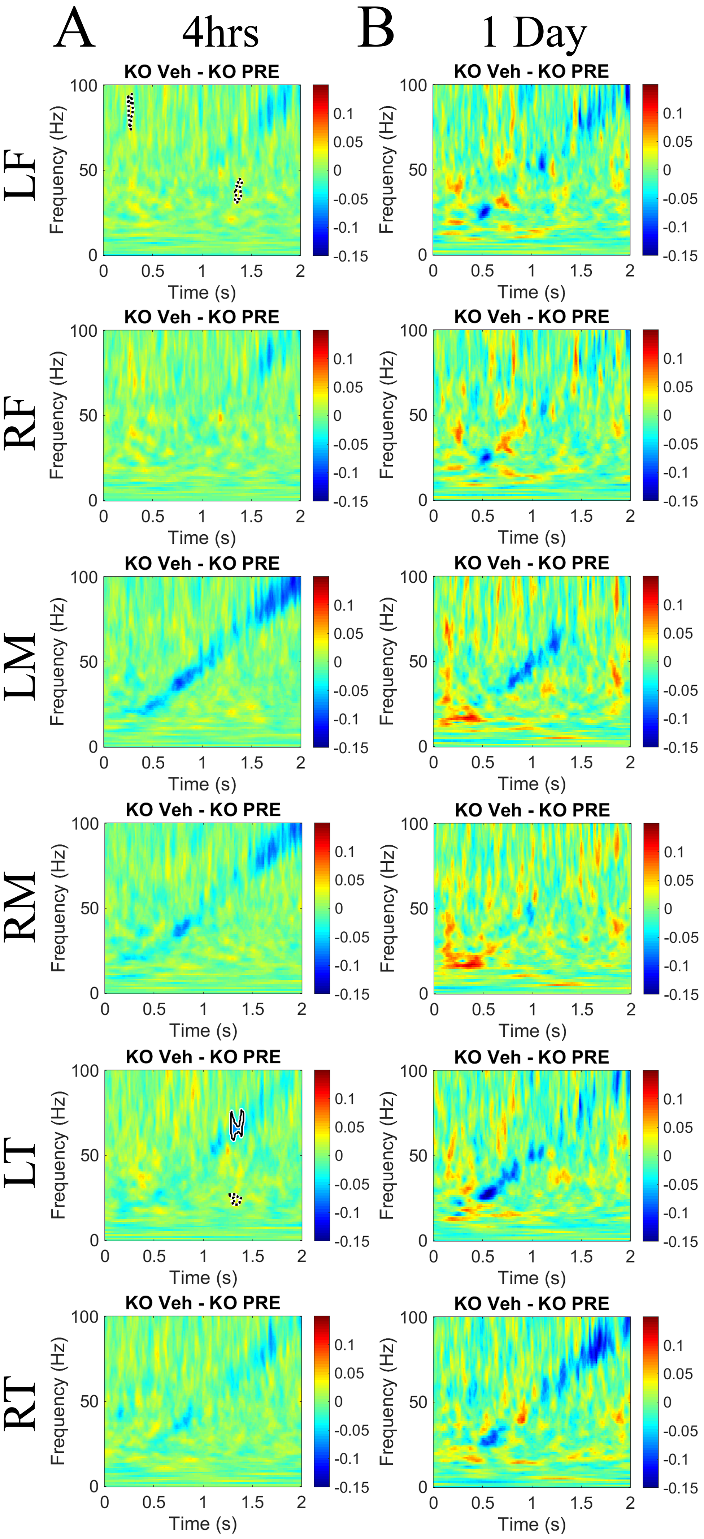


**Supplementary Figure S4.4. Chirp ITPC in vehicle treated *Fmr1* KO mice compared to controls.** A-B) Graphs show ITPC difference between 4 hour and 1-day vehicle treated *Fmr1* KO mice (4h, N=7; 1d, N=2) and *Fmr1* KO mice prior to treatment (PRE, N=22). Abbreviations: left/right frontal (LF/RF), left/right medial (LM/RM), left/right temporal (LT/RT).


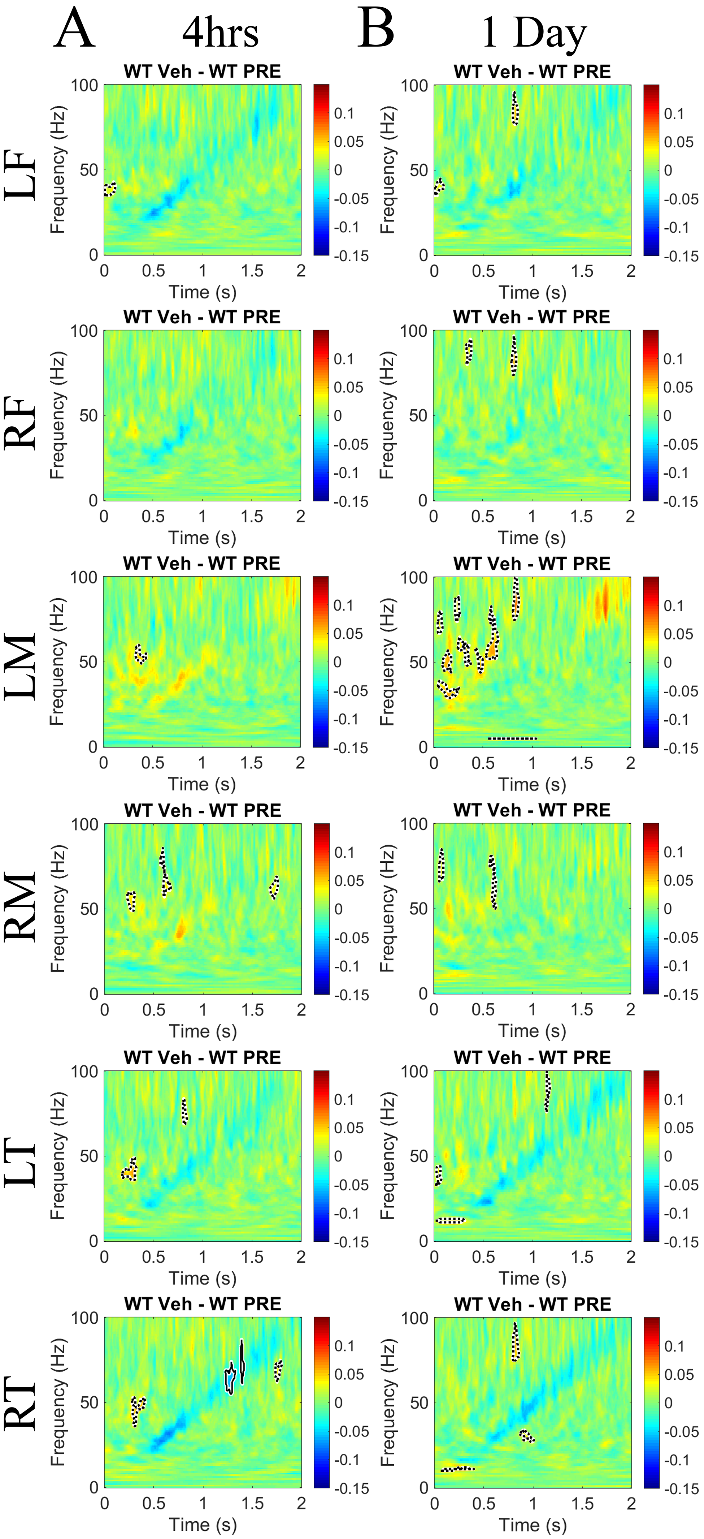


**Supplementary Figure S4.5. Chirp ITPC in vehicle treated WT mice compared to controls.** A) Graphs show ITPC difference between 4 hour and 1-day vehicle treated WT mice (4h, N=10; 1d, N=10) and WT mice prior to treatment (PRE, N=24). Abbreviations: left/right frontal (LF/RF), left/right medial (LM/RM), left/right temporal (LT/RT).

**Supplementary Table S1. Statistical results for eCB lipid levels in the auditory cortex of *Fmr1* KO and WT mice 4h post treatment (Figure 1). All analysis was performed using two-way ANOVA.**

|  | **Genotype** | **Treatment** | **Interaction** |
| --- | --- | --- | --- |
| **2-AG** | F(1,53)=1.79, p=0.19 | F(3,53)=62.38, p<0.0001 | F(3,53)=0.64, p=0.59 |
| **DHAG** | F(1,52)=0.66, p=0.42 | F(3,52)=28.21, p<0.0001 | F(3,52)=1.64, p=0.19 |
| **2-OG** | F(1,53)=0.80, p=0.38 | F(3,53)=4.08, p=0.01 | F(3,53)=1.25, p=0.30 |
| **2-LG** | F(1,53)=0.23, p=0.63 | F(3,53)=3.26, p=0.03 | F(3,53)=1.09, p=0.36 |
| **AEA** | F(1,51)=7.18, p=0.0099 | F(3,51)=7.66, p=0.0003 | F(3,51)=4.41, p=0.008 |
| **OEA** | F(1,52)=11.01, p=0.002 | F(3,52)=10.07, p<0.0001 | F(3,52)=1.42, p=0.25 |
| **DHEA** | F(1,53)=3.41, p=0.07 | F(3,53)=5.79, p=0.002 | F(3,53)=1.01, p=0.40 |

**Supplementary Table S2. Statistical results for eCB lipid levels in the auditory cortex of *Fmr1* KO and WT mice 1d post treatment (Figure S1). All analysis was performed using two-way ANOVA.**

|  | **Genotype** | **Treatment** | **Interaction** |
| --- | --- | --- | --- |
| **2-AG** | F(1,13)=0.68, p=0.43 | F(1,13)=2.82, p=0.12 | F(1,13)=0.13, p=0.73 |
| **DHAG** | F(1,13)=5.33, p=0.04 | F(1,13)=0.11, p=0.75 | F(1,13)=0.11, p=0.75 |
| **2-OG** | F(1,13)=4.19, p=0.06 | F(1,13)=0.07, p=0.79 | F(1,13)=0.02, p=0.89 |
| **2-LG** | F(1,13)=5.61, p=0.03 | F(1,13)=0.01, p=0.90 | F(1,13)=0.24, p=0.63 |
| **AEA** | F(1,13)=1.34, p=0.27 | F(1,13)=0.18, p=0.68 | F(1,13)=2.33, p=0.15 |
| **OEA** | F(1,12)=2.57, p=0.13 | F(1,12)=1.62, p=0.23 | F(1,12)=2.13, p=0.17 |
| **DHEA** | F(1,12)=1.28, p=0.28 | F(1,12)=2.97, p=0.11 | F(1,12)=3.76, p=0.08 |

**Supplementary Table S3. Statistical results for spectral power comparisons of JZL-184 treated *Frm1* KO mice 4h and 1d post treatment compared to *Fmr1* KO mice prior to treatment in Left Frontal, Right Frontal, Left Medial, Right Medial, Left Temporal, and Right Temporal regions (Figure 3).**

|  | **Treatment** | **Frequency** | **Interaction** |
| --- | --- | --- | --- |
| **Left Frontal** | F (2, 240) = 3.42,  p=0.03 | F (5, 240) = 0.176,  p=0.971 | F (10, 240) = 0.216,  p=0.99 |
| **Right Frontal** | F (2, 240) = 6.75,  p=0.0014 | F (5, 240) = 0.264,  p=0.933 | F (10, 240) = 0.185,  p=0.99 |
| **Left Medial** | F (2, 240) = 0.784,  p=0.458 | F (5, 240) = 0.389,  p=0.856 | F (10, 240) = 0.221,  p=0.99 |
| **Right Medial** | F (2, 240) = 7.75,  p=0.0005 | F (5, 240) = 0.290,  P=0.918 | F (10, 240) = 0.156,  P=0.99 |
| **Left Temporal** | F (2, 240) = 2.99,  P=0.05 | F (5, 240) = 0.226,  P=0.951 | F (10, 240) = 0.172,  P=0.99 |
| **Right Temporal** | F (2, 240) = 10.78,  P<0.0001 | F (5, 240) = 0.119,  P=0.988 | F (10, 240) = 0.118,  P=0.99 |

**Supplementary Table S4A-B. Power coupling comparison of alpha vs. low gamma and theta vs. low gamma in six regions.** Values in table represent P values. Statistically significant p values are bolded (*, p<0.05; **, p<0.01; ***, p<0.001; ****, p<0.0001). For alpha- and theta-low gamma Pearson’s correlation, student’s t-test were used to compare treatment (8mg/kg JZL-184 x vehicle) groups. Abbreviations: Left Frontal (1), Right Frontal (2), Left Medial (3), Right Medial (4), Left Temporal (5), and Right Temporal (6).

**Supplementary Table S4A. KO PRE vs. KO JZL (4h)**

|  | **G1** | **G2** | **G3** | **G4** | **G5** | **G6** |
| --- | --- | --- | --- | --- | --- | --- |
| **A1** | **0.0146** | 0.0534 | 0.0876 | 0.1869 | 0.6051 | 0.5857 |
| **A2** | **0.044** | 0.1229 | 0.1951 | 0.2787 | 0.5633 | 0.6063 |
| **A3** | 0.1719 | 0.5549 | 0.9886 | 0.9484 | 0.718 | 0.8902 |
| **A4** | 0.2158 | 0.4832 | 0.8547 | 0.751 | 0.6341 | 0.797 |
| **A5** | 0.0545 | 0.0875 | 0.1513 | 0.1491 | 0.3533 | 0.2605 |
| **A6** | **0.0341** | 0.0681 | 0.1073 | 0.1935 | 0.193 | 0.3054 |
| **T1** | **0.0007** | **0.0007** | **0.0104** | **0.0058** | **0.0003** | **0.0025** |
| **T2** | **0.0005** | **0.0005** | **0.0350** | **0.0051** | **0.0107** | **0.0025** |
| **T3** | **0.0083** | **0.0093** | **0.0463** | **0.0313** | **0.0311** | **0.0019** |
| **T4** | **0.0051** | **0.0023** | **0.0355** | **0.0183** | **0.0018** | **0.0066** |
| **T5** | **0.0017** | **0.0025** | **0.0380** | **0.0095** | **0.0257** | **0.0077** |
| **T6** | **0.0002** | **0.0003** | **0.0067** | **0.0016** | **0.0024** | **0.0009** |

**Supplementary Table S4B. KO PRE vs. KO JZL (1d)**

|  | **G1** | **G2** | **G3** | **G4** | **G5** | **G6** |
| --- | --- | --- | --- | --- | --- | --- |
| **A1** | 0.0664 | 0.0868 | 0.2867 | 0.7097 | 0.3544 | 0.3184 |
| **A2** | 0.1086 | 0.0983 | 0.3077 | 0.7934 | 0.0601 | 0.4168 |
| **A3** | **0.0249** | **0.0199** | **0.0241** | **0.0346** | **0.0453** | **0.0155** |
| **A4** | **0.014** | **0.0194** | **0.0279** | **0.0537** | **0.0378** | **0.0234** |
| **A5** | 0.3374 | 0.2872 | 0.1476 | 0.7275 | 0.5389 | 0.4783 |
| **A6** | 0.2402 | 0.1868 | 0.0622 | 0.5391 | 0.4232 | 0.3709 |
| **T1** | 0.3245 | 0.3043 | 0.1927 | 0.1188 | 0.0367 | 0.2743 |
| **T2** | 0.2974 | 0.3344 | 0.1599 | 0.1241 | 0.0982 | 0.2711 |
| **T3** | 0.8755 | 0.7116 | 0.7498 | 0.7859 | 0.7107 | 0.909 |
| **T4** | 0.9366 | 0.8851 | 0.6202 | 0.7148 | 0.3676 | 0.9363 |
| **T5** | 0.374 | 0.4956 | 0.3646 | 0.2226 | 0.2054 | 0.3192 |
| **T6** | 0.3945 | 0.4922 | 0.3675 | 0.2985 | 0.1345 | 0.3842 |

**Supplementary Table S5. Statistical results for locomotor activity, anxiety, and hyperactivity measures of WT and *Fmr1* KO mice following open field (OF) and elevated plus maze (EPM) tests (Figure 6). All analysis was performed using two-way ANOVA.**

|  | **Genotype** | **Treatment** | **Interaction** |
| --- | --- | --- | --- |
| **Defecation (OF)** | F (1, 49) = 5.323,  p=0.025 | F (1, 49) = 6.327,  p=0.015 | F (1, 49) = 3.814,  p=0.057 |
| **Speed (mm/sec) (OF)** | F (1, 49) = 3.656,  p=0.062 | F (1, 49) = 11.83,  p=0.0012 | F (1, 49) = 0.411,  p=0.525 |
| **Stretch-attend posture (OF)** | F (1, 49) = 1.197,  p=0.279 | F (1, 49) = 12.66,  p=0.0008 | F (1, 49) = 1.158,  p=0.287 |
| **Time in center per entry (OF)** | F (1, 48) = 0.525,  p=0.472 | F (1, 48) = 4.43,  p=0.041 | F (1, 48) = 4.361,  p=0.042 |
| **Total distance (OF)** | F (1, 49) = 2.81,  p=0.099 | F (1, 49) = 12.08,  p=0.001 | F (1, 49) = 0.237,  p=0.629 |
| **Total entries (OF)** | F (1, 49) = 2.056,  p=0.158 | F (1, 49) = 10.87,  p=0.002 | F (1, 49) = 0.309,  p=0.581 |
| **Stretch-attend posture (EPM)** | F (1, 49) = 0.725,  p=0.399 | F (1, 49) = 35.06,  P<0.0001 | F (1, 49) = 0.115,  p=0.736 |
| **Grooming bouts (EPM)** | F (1, 49) = 0.582,  P=0.449 | F (1, 49) = 1.69,  P=0.199 | F (1, 49) = 0.015,  P=0.904 |
| **% time in open arms (OA)** | F (1, 49) = 5.22,  P=0.027 | F (1, 49) = 5.08,  P=0.029 | F (1, 49) = 0.012,  P=0.912 |

**Supplementary Table S6. Summary table showing locomotor activity, anxiety, and hyperactivity measures for WT and *Fmr1* KO mice during the open field (OF) and elevated plus maze (EPM) tests shown in Figure 6 (mean ± SEM).**

|  | **WT Vehicle** | **WT JZL-184** | **KO Vehicle** | **KO JZL-184** |
| --- | --- | --- | --- | --- |
| **Defecation (OF)** | 0.29 ± 0.11 | 0.1 ± 0.1  ** (p=0.007)  KO veh | 1.76 ± 0.48  ** (p=0.005) | 0.22 ± 0.22  * (p=0.02) |
| **Speed (mm/sec) (OF)** | 46.95 ± 2.36 | 39.39 ± 3.12  ** (p=0.002)  KO veh | 53.84 ± 2.32 | 42.82 ± 2.61  * (p=0.04) |
| **Stretch-attend posture (OF)** | 3.65 ± 0.56 | 2.1 ± 0.48  ** (p=0.008)  KO veh | 5.00 ± 0.61 | 2.11 ± 0.59  * (p=0.01) |
| **Time in center per entry (OF)** | 0.63 ± 0.04 | 0.63 ± 0.05 | 0.56 ± 0.04 | 0.76 ± 0.06  * (p=0.03) |
| **Total distance (OF)** | 13383.79 ± 630.70 | 11272.33 ± 853.38  ** (p=0.003)  KO veh | 14912.32 ± 573.87 | 12113.32 ± 690.04  * (p=0.04) |
| **Total entries (OF)** | 283.53 ± 9.51 | 249.40 ± 17.11  ** (p=0.007)  KO veh | 308.29 ± 8.15 | 260.33 ± 18.00  * (p=0.04) |
| **Stretch-attend posture (EPM)** | 20.24 ± 1.10 | 10.89 ± 1.10  **** (p<0.0001)  KO veh; *** (p=0.003)  WT veh | 21.00 ± 1.57 | 12.67 ± 1.31  ** (p=0.001) KO veh; ** (p=0.004) WT veh |
| **Grooming bouts (EPM)** | 1.65 ± 0.37 | 1.11 ± 0.26 | 1.89 ± 0.31 | 1.44 ± 0.38 |
| **% time in open arms (OA)** | 28.05 ± 2.53 | 21.74 ± 3.29  * (p=0.01)  KO veh | 33.85 ± 1.95 | 28.13 ± 2.52 |
